# Plasmonic twinned silver nanoparticles with molecular precision

**DOI:** 10.1038/ncomms12809

**Published:** 2016-09-09

**Authors:** Huayan Yang, Yu Wang, Xi Chen, Xiaojing Zhao, Lin Gu, Huaqi Huang, Juanzhu Yan, Chaofa Xu, Gang Li, Junchao Wu, Alison J. Edwards, Birger Dittrich, Zichao Tang, Dongdong Wang, Lauri Lehtovaara, Hannu Häkkinen, Nanfeng Zheng

**Affiliations:** 1 Collaborative Innovation Center of Chemistry for Energy Materials, State Key Laboratory for Physical Chemistry of Solid Surfaces, and Engineering Research Center for Nano-Preparation Technology of Fujian Province, College of Chemistry and Chemical Engineering, Xiamen University, 422 Siming South Road, Xiamen 361005, China; 2 Nanoscience Center, Department of Chemistry, University of Jyväskylä, Box 35, 40014 Jyväskylä, Finland; 3 Institute of Physics, Chinese Academy of Sciences, Beijing 100190, China; 4 State Key Laboratory of Molecular Reaction Dynamics, Dalian Institute of Chemical Physics, Chinese Academy of Sciences, Dalian 116023, China; 5 Department of Civil Engineering, Xiamen University, Xiamen, Fujian 361005, China; 6 Australian Nuclear Science and Technology Organization, Australian Centre for Neutron Scattering, New Illawarra Road, Lucas Heights, New South Wales 2234, Australia; 7 Heinrich-Heine Universität Düsseldorf, Anorganische Chemie und Strukturchemie, Universitätsstrasse 1, Gebäude 26.42.01.21, 40225 Düsseldorf, Germany; 8 Nanoscience Center, Department of Physics, University of Jyväskylä, Box 35, 40014 Jyväskylä, Finland

## Abstract

Determining the structures of nanoparticles at atomic resolution is vital to understand their structure–property correlations. Large metal nanoparticles with core diameter beyond 2 nm have, to date, eluded characterization by single-crystal X-ray analysis. Here we report the chemical syntheses and structures of two giant thiolated Ag nanoparticles containing 136 and 374 Ag atoms (that is, up to 3 nm core diameter). As the largest thiolated metal nanoparticles crystallographically determined so far, these Ag nanoparticles enter the truly metallic regime with the emergence of surface plasmon resonance. As miniatures of fivefold twinned nanostructures, these structures demonstrate a subtle distortion within fivefold twinned nanostructures of face-centred cubic metals. The Ag nanoparticles reported in this work serve as excellent models to understand the detailed structure distortion within twinned metal nanostructures and also how silver nanoparticles can span from the molecular to the metallic regime.

## Abstract (web summary)

The structure of nanoparticles strongly influences their properties. Here, the authors use single crystal X-ray diffraction to resolve the crystal structures of Ag_136_ and Ag_374_ nanoparticles, enabling the observation of local structure distortion and the lower size limit of surface plasmon resonance.

## Graphical abstract

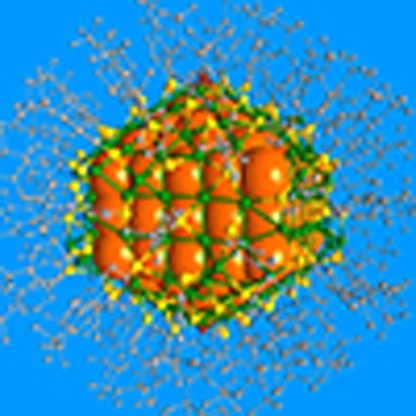

Owing to quantum-size and surface effects, nanoparticles frequently exhibit novel physical and chemical properties, which differ from their bulk counterparts in dramatic ways, and they have been gaining increasing attention in the fundamental and applied sciences^1^,^2^,^3^,^4^,^5^,^6^,^7^. Properties of nanoparticles are governed by chemical composition, size, shape and the overall molecular structure^1^,^2^. Despite significant progress in the chemical fabrication of uniform functional nanoparticles with well-defined shapes, sizes and compositions over the past two decades^8^,^9^,^10^,^11^, it is still notoriously difficult to manipulate their structures and thus functions with molecular control. Currently, there is the lack of effective tools to characterize the detailed structures (for instance, defects, twinning and surface features) of functional nanoparticles at atomic resolution. Without a detailed molecular structure as a guide, precision synthesis of nanoparticles with targeted functionality is difficult.

During the last several years, two major strategies have been applied in pursuit of detailed structure analysis of metal nanoparticles. One is application of state-of-the-art electron microscopy techniques to probe the atomic-resolution structures of the nanoparticle core^12^,^13^,^14^,^15^,^16^,^17^. Azubel *et al*.^16^ have reported the structure determination of a ligand-stabilized Au_68_ nanoparticle at atomic resolution by a combination of a low-dose approach and aberration-corrected transmission electron microscopy (TEM). The other avenue is to prepare and crystallize monodisperse metal nanoparticles into single crystals and determine their structures using X-ray diffraction^18^,^19^,^20^,^21^,^22^,^23^,^24^,^25^,^26^,^27^,^28^,^29^,^30^,^31^. Such an approach is particularly important to resolve the metal-ligand interfacial features at the metal nanoparticle surface. By this approach, the structures of a few ligand-stabilized (for instance, carbon monoxide and thiolate) metal nanoparticles containing over 100 metal atoms (for instance, Au_102_, Au_133_ and Au_130_) have been determined at or near atomic resolution^18^,^19^,^20^,^21^,^32^. Despite significant progress following both strategies, resolution of the molecular structure of metal nanoparticles containing several hundred metal atoms and having metallic properties remains daunting^20^,^33^.

We report here the syntheses and structure determinations of two giant, thiolated Ag nanoparticles containing 136 and 374 Ag atoms. As the largest thiolated metal nanoparticles crystallographically determined so far, these Ag nanoparticles exhibit unprecedented metallic properties with the emergence of surface plasmon resonance (SPR). The nanoparticles are miniatures of two closely related fivefold twinned nanostructures, pentagonal-bipyramidal (that is, decahedral) nanoparticles and twinned nanorods/nanowires derived from decahedral particles by elongation along the fivefold axis. Small distortions from the structure archetype within the fivefold twinned nanostructures are observed. Density functional theory (DFT) studies reveal that the smaller nanoparticle has molecular character with a small but distinct energy gap (band gap) between occupied and unoccupied orbitals (highest occupied molecular orbital–lowest unoccupied molecular orbital gap), whereas the larger one is fully metallic without a band gap. This leads to emergence of the surface plasmon incorporating contributions from the organic ligand layer. These two structurally determined systems represent important exemplars of the cross-over of Ag nanoparticles from the molecular to the metallic regime.

## Results

### Syntheses and TEM characterizations

**Figure 1:**
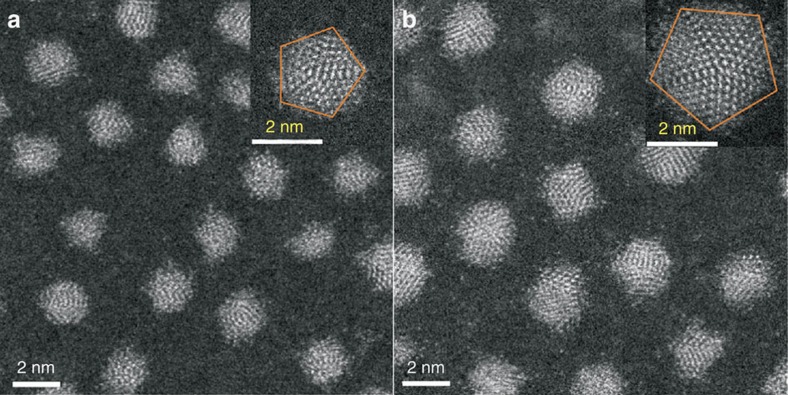
Electron micrographs of thiolated Ag nanoparticles. Scanning TEM and high-resolution TEM (inset) images of the as-prepared small (**a**) and large (**b**) 4-*tert*-butylbenzenethiolate-stabilized Ag nanoparticles. Scale bars, 2 nm.

The syntheses of thiolated Ag nanoparticles were achieved by chemical reduction of a polymeric silver 4-*tert*-butylbenzenethiolate precursor by NaBH_4_ in the presence of PPh_4_Br and triethylamine (see Methods for full details). The resulting Ag nanoparticles were first characterized by TEM as shown in Fig. 1a and Supplementary Fig. 1. The TEM images revealed that these thiolated Ag nanoparticles had a tight particle-size distribution at ∼2 nm. Thiolated Ag nanoparticles of larger, likewise uniform size, at ∼3 nm (Fig. 1b and Supplementary Fig. 2) were prepared in a similar manner, by increasing the Ag:SR (thiolate) molar ratio to 1.4:1.

Two important features are associated with these thiolated Ag nanoparticles. First, their sizes lie in the region where metallic Ag nanoparticles are said to develop SPR^34^ and thus provide scope to assess the occurrence of SPR from the viewpoint of associated quantum mechanical calculations. Second, the small and large Ag nanoparticles are verified as fivefold twinned nanocrystals as initially revealed by high-resolution TEM analysis (insets in Fig. 1 and Supplementary Figs 3 and 4). Fivefold twinning is a common phenomenon for nanoparticles of face-centred cubic (fcc) metals^9^. Competing models for the internal structure of fivefold twinned nanoparticles have been debated in the literature^35^,^36^. Resolving these atomic scale structures now demonstrates at the molecular level how the lattice mismatches are readily resolved within real fivefold twinned nanoparticles.

### Molecular structures from single-crystal X-ray diffraction

**Figure 2:**
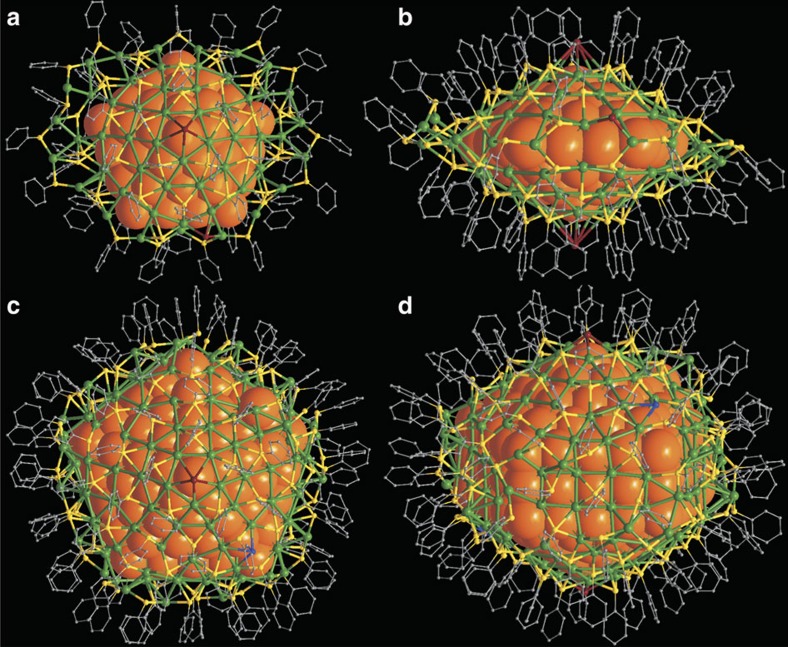
The overall structures of Ag_136_ and Ag_374_ nanoparticles. (**a**,**b**) Top and side views of [Ag_136_(SR)_64_Cl_3_Ag_0.45_]^−^. (**c**,**d**) Top and side views of [Ag_374_(SR)_113_Br_2_Cl_2_]. Colour legend: orange, core Ag; green, surface Ag; yellow, S; brown, halogen; blue, Cl; grey, C. All hydrogen atoms and *tert*-butyl groups are omitted for clarity.

Encouraged by the uniform size of both the small and large thiolated Ag nanoparticles, crystallization of these compounds was undertaken. High-quality black prism crystals of small nanoparticles and block crystals of large nanoparticles that were suitable for X-ray analysis were obtained by diffusion of hexane into their dichloromethane solutions (Supplementary Fig. 5). The fivefold twinned feature of both small and large silver nanoparticles was indeed revealed by detailed single crystal X-ray diffraction studies (for 1.2 Å resolution data in each case; Supplementary Data 1 and 2). Initial structure solutions were acquired by means of SHELXL^37^ and model refinements combined techniques of macromolecular crystallography with those more typical of chemical crystallography (Supplementary Methods and Supplementary Tables 1 and 2).^38^,^39^,^40^,^41^,^42^ As shown in Fig. 2, the small nanoparticle has a composition modelled as [Ag_136_(SR)_64_Cl_3_Ag_0.45_]^−^ (denoted as Ag_136_) with the apparent mono-anionic charge balanced by a well-resolved PPh_4_^+^ and one entire nanoparticle plus cation pair forming the asymmetric unit. The larger nanoparticle has a composition modelled as [Ag_374_(SR)_113_Br_2_Cl_2_] (denoted as Ag_374_) with one half molecule lying on a crystallographic twofold axis comprising the asymmetric unit (Supplementary Figs 6 and 7). SR in both cases is *4-tert*-butylthiophenolate. The presence of Cl in Ag_136_, and both Cl and Br in Ag_374_ were confirmed by temperature-programmed decomposition/mass-spectrometric and electrospray ionization mass-spectrometric data (Supplementary Fig. 8). Ag_136_ and Ag_374_ represent the largest thiolated metal nanoparticles with molecular structures determined by single-crystal X-ray analysis. They bear no structural relationship to a previously reported series of thiolated Ag_*x*_S_*y*_ particles containing up to 490 Ag atoms, derived from bulk Ag_2_S semiconducting materials^43^,^44^.

**Figure 3:**
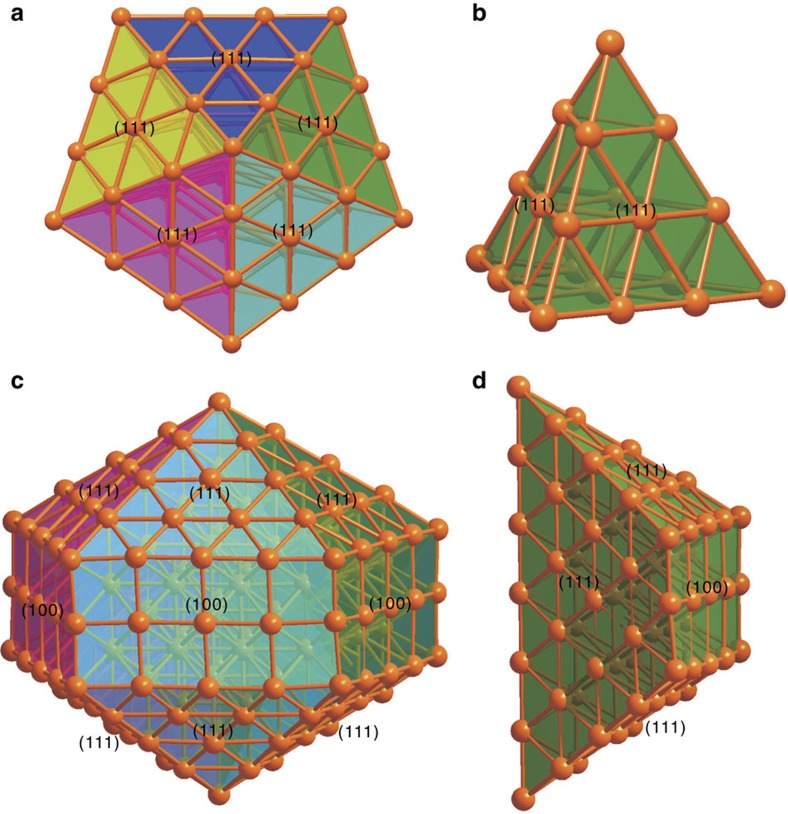
The structure dissection of the fivefold twinned cores of Ag_136_ and Ag_374_ nanoparticles. (**a**) Core of Ag_136_, consisting of five tetrahedral units (**b**). (**c**) Core of Ag_374_, consisting of five wedge-shaped units (**d**). Different colours are used to highlight the five different twinning domains of the cores.

Both Ag_136_ and Ag_374_ can be structurally described as a fivefold twinned core enclosed within related structurally distinctive Ag–SR complex shells (Fig. 2). While the fivefold twinned core in Ag_136_ is present as a pentagonal bipyramid of 54 Ag atoms, the core in Ag_374_ is an elongated pentagonal bipyramid (Ino's decahedron) consisting of 207 Ag atoms. The Ag_54_ core in Ag_136_ can be structurally described as five conjoined tetrahedral domains of fcc Ag (Fig. 3a), each of which consists of 20 Ag atoms (Fig. 3b) and having 4 external Ag {111} facets. Each tetrahedral subunit is joined to the adjacent tetrahedral subunits by sharing of common triangular faces. In comparison, the Ag_207_ core of Ag_374_ can be considered as a miniature fivefold twinned nanorod constructed from five conjoined single-crystalline wedge-shaped grains, which are related to the pentagonal-bipyramid by elongation of each constituent tetrahedron to a wedge along the molecular fivefold aspect (Fig. 3c). In each wedge-shaped grain, there are two (111) triangular faces at the apices, two (111) trapezoids with their long edge on the fivefold axis and one (100) rectangle forming each side (Fig. 3d). Overall, each nanorod has five (100) side surfaces parallel to the elongated direction and ten (111) surfaces at the apices of the rod. The 207 Ag atoms in the core are distributed about a central Ag atom successively surrounded by three elongated pentagonal bipyramids consisting of 12, 42 and 92 atoms, encircled by a ‘pentagonal cylinder' of 60 Ag atoms likewise centred about the fivefold axis of the nanorod (Supplementary Fig. 9). The metal distributions in the cores of both Ag_136_ and Ag_374_ are entirely distinct from that in Au_133_ whose structure was reported (after submission of this report) to have a 20-fold twinned icosahedral core^20^,^21^. It should be noted that fivefold twinned metal cores have also been previously observed in Au_102_ and Au_130_ clusters resolved by single-crystal X-ray analysis^19^,^32^ and Au_309_ characterized by aberration-corrected scanning TEM^13^.

### Structural distortions inside fivefold twinned cores

In regular fcc metals, the idealized angle between two (111) faces is 70.53°. When joined together by sharing (111) faces along the fivefold axis, without distortion, five ideal single-crystalline grains in a fivefold twinned nanostructure can only subtend an angle of 352.65°, 7.35° short of closure (Supplementary Fig. 10)^17^,^45^. In a real fivefold twinned nanostructure, this solid-angle deficiency needs to be compensated by sufficiently adjusting the interatomic spacings, introduction of various defects such as dislocations and stacking faults, or by increased internal vibrations. Structural models with homogeneous or inhomogeneous strain (deviations from idealized values) have been proposed for small metal nanoparticles^35^,^36^. In the fivefold twinned cores of Ag_136_ and Ag_374_, the average Ag–Ag bond lengths are 2.870 and 2.882 Å, respectively, both slightly shorter than the Ag–Ag bond distance (2.889 Å) of bulk silver with Ag_374_ only marginally so.

Detailed review of the Ag–Ag bond lengths (Supplementary Fig. 11) demonstrates the presence of a distinctive distortion within the observed pentagonal bipyramidal Ag_54_ core, where an obvious anisotropy of these adjustments is clear. Although Ag–Ag distances (18 out of 23) parallel to the fivefold axis are shorter than the average Ag–Ag distance (2.870 Å), most Ag–Ag bonds (47 out of 50) perpendicular to the fivefold axis are longer than 2.870 Å. Such a distribution clearly suggests that the geometric non-ideality in the decahedral core is resolved via a modest compression along the fivefold axis and concomitant relaxation about the directions perpendicular to the fivefold axis. In other words, each tetrahedral grain in the fivefold twinned decahedral nanoparticle is slightly compressed along the molecular fivefold axis. In Ag_136_, the five relevant angles are 71.5, 71.7, 72.1, 72.3 and 72.5°, slightly expanded from the idealized 70.53° by 1°–2°. These small deviations eliminate the 7.35° deficiency. Geometrically, this slight anisotropy evidently minimizes the total potential energy of the silver core (Supplementary Figs 12–14). In contrast to the anisotropy observed in the Ag_54_ core, no obvious trends are seen in the Ag_207_ core. Small deviations from planarity (a slight bulging) at the twinning boundaries appears to be the only readily discernible compensation for the solid-angle deficiency of the fivefold twinned Ag_207_ core. Careful analysis reveals that the fivefold twinning boundary (111) faces in the Ag_207_ core are not strictly planar (Supplementary Fig. 15). Within each shared (111) face, some Ag atoms deviate from the plane of their coplanar Ag set by up to 0.20 Å.

### Surface structures

**Figure 4:**
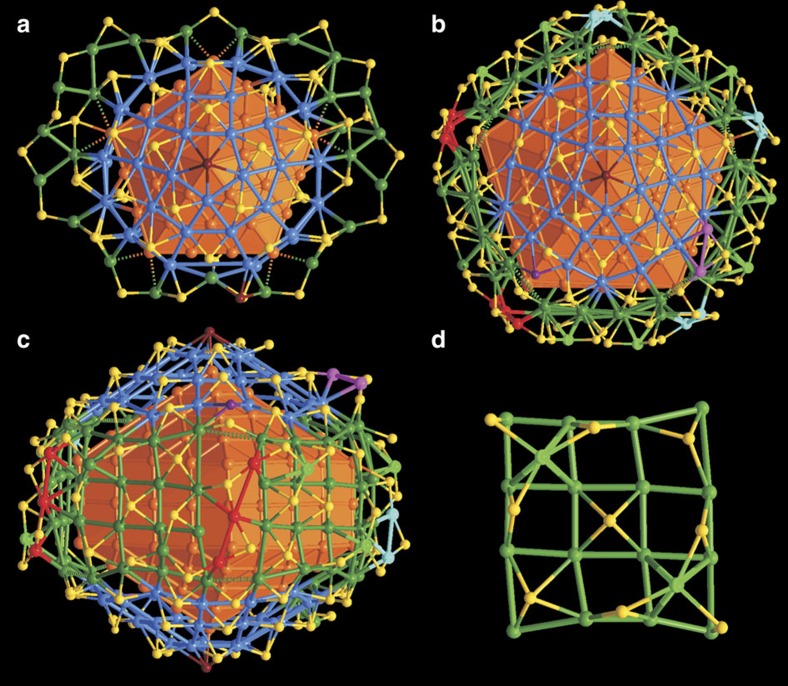
The surface structures of Ag_136_ and Ag_374_ nanoparticles. (**a**) Top view of the complex shell of Ag_136_ with the bowl-like half J73 related [Ag_30_(SR)_15_Cl] caps highlighted in blue. (**b**,**c**) Top and side views of the complex shell of Ag_374_ with key structure elements highlighted in different colours. (**d**) Representative 4 × 4 arrangement of surface Ag atoms on (100) side surface of the Ag_207_ core. Colour legend: yellow sphere, S; brown sphere, halogen; the rest, Ag.

Based on TEM measurements, many studies have concluded that a decahedral nanoparticle should be bound by ten (111) facets and a fivefold twinned nanorod/nanowire of metals should have five (100) faces at its side and ten (111) faces, five at either end^9^. The X-ray studies of Ag_136_ and Ag_374_ validate this hypothesis for their Ag_54_ and Ag_207_ cores. However, the crystallographic studies reveal that the thiolate capped outermost Ag atoms deviate from the close-packed archetypes in a significant manner but with a marked congruence of the deviations from close packing observed in the two structures. In Ag_136_, the Ag_54_ core comprises two pentagonal pyramids, each of which is surmounted by a bowl-like [Ag_30_(SR)_15_Cl] unit (Fig. 4a). The silver atoms in [Ag_30_(SR)_15_Cl] describe one half of a parabigyrate rhombicosidodecahedron (Johnson solid J73—a circumscribable 60 vertex figure). These 30 silver atoms lie with their fivefold aspect disposed about the same fivefold axis as the Ag_54_ core and the opposing Ag_30_ unit lies in an eclipsed configuration about the fivefold core aspect. With each apical pentagon surmounted by a Cl^−^ anion, each of the other distorted pentagonal Ag_5_ and tetragonal Ag_4_ faces is capped by a thiolate ligand. The equatorial pentagon of the Ag_54_ core is encircled by a Ag–SR complex ring. Four of the equatorial corners are spanned by one of four Ag_2_(SR)_5_ units and the remaining corner is spanned by one Ag_2_(SR)_4_Cl unit (against which the PPh_4_^+^ counterion rests). Detailed geometric analysis reveals that two significantly larger Ag–SR–Ag bond angles (135.8(4)° and 134.2(8)°) occur within these units than in the remainder (107.1(7)°, 108.5(3)° and 105.8(5)° for Ag_2_(SR)_4_Cl; Supplementary Fig. 16). Each of those with the larger Ag–SR–Ag bond angles is further spanned by Ag_6_(SR)_5_ units. These units connect via Ag and S to the units they span and bridge via their Ag termini between the two smaller angle Ag_2_(SR)_5_ and Ag_2_(SR)_4_Cl completing the outer AgS layer.

In a strikingly similar manner, the fivefold twinned Ag_207_ core of Ag_374_ is also fully encapsulated by a complex Ag-thiolate layer. As shown in Fig. 4b, each pentagonal pyramid is analogously capped by a bowl-like [Ag_30_(SR)_15_Br] of similar half J73 configuration with the apical pentagonal sites occupied by bromide. The five side (100) faces of the Ag_207_ core are each covered by five near planar Ag_16_ units. In each Ag_16_ unit, the silver atoms are arranged in somewhat irregular 4 × 4 patterns (Fig. 4c,d). The atoms in each Ag_16_ unit are face-capped by three SR and two Ag(SR)_3_ motifs (Supplementary Fig. 17). At the five pentagonal prismatic edges of the Ag_207_ core, the Ag_16_ units are joined together by three Ag_3_(SR)_2_, two Ag_2_(SR)_2_ motifs and four bridging SR, forming a drum-like layer surrounding the elongated pentagonal prismatic equatorial aspect of the core. This drum-like layer connects the two bowl-like [Ag_30_(SR)_15_Br] units via shared thiolates of the drum and a further two groups of Ag_2_(SR)_2_ motifs, and ten bridging SR and one Cl, which completes the closed complex shell having an overall composition of [Ag_167_(SR)_113_Cl_2_Br_2_] (Supplementary Fig. 18) surrounding the Ag_207_ core. For both Ag_136_ and Ag_374_, the Ag_30_ half-J73 domes lie with their apical pentagons eclipsed rather than staggered (as occurs in a complete 60 vertex J73 figure).

### Electronic structures and optical properties

**Figure 5:**
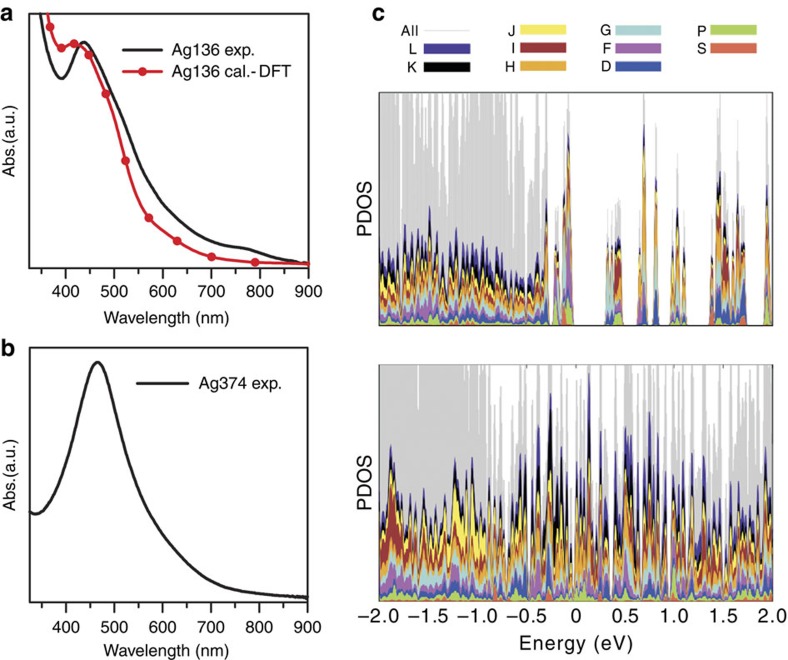
Optical properties and electronic structures of Ag_136_ and Ag_374_ nanoparticles. (**a**,**b**) Ultraviolet–visible absorption spectra of (**c**) Ag_136_ (experimental and computed) and (**b**) Ag_374_ (experimental) nanoparticles. In the calculated spectra, the individual transitions are smoothed by using a Gaussian width of 0.1 eV. (**c**) Projection of the Kohn–Sham electron states (projected densities of electron states (PDOS)) of Ag_136_ (top) and Ag_374_ (bottom) to spherical harmonics centred at the metal core. The different spherical harmonics components (S, P, D,...) are indicated with colours as shown in the legend. The Fermi energy is at zero.

Both thiolated Ag_136_ and Ag_374_ nanoparticles are readily dissolved in solvents such as chloroform and dichloromethane, to give brown solutions. As shown in Fig. 5a,b, the smaller Ag_136_ nanoparticles display a broad major peak centred around 450 nm (2.75 eV) and a weak shoulder peak at 772 nm, whereas the larger Ag_374_ nanoparticles show only one strong peak at 465 nm (2.67 eV). The Ag_374_ absorption behaviour is distinct from the molecule-like multiband absorption features of previously reported thiolated Ag nanoclusters (for instance, Ag_14_, Ag_16_, Ag_25_, Ag_32_ and Ag_44_)^22^,^23^,^46^,^47^,^48^,^49^,^50^ and that of Ag_136_.

The electronic structures of Ag_136_ and Ag_374_ were probed via DFT computations, by using the simplified SPh ligand in place of SPh-*t*Bu for Ag_136_ with a further simplification to SH for Ag_374_. The projected densities of electron states of the clusters are shown in Fig. 5c and Supplementary Fig. 19. The calculated highest occupied molecular orbital–lowest unoccupied molecular orbital band gap for Ag_136_ is 0.37 eV, whereas the band gap is closed for Ag_374_. The calculated band gap and angular momentum characteristics of the frontier orbitals (Supplementary Fig. 20) place Ag_136_ as a molecular system, whereas the Ag_374_ can be characterized as metallic.

**Figure 6:**
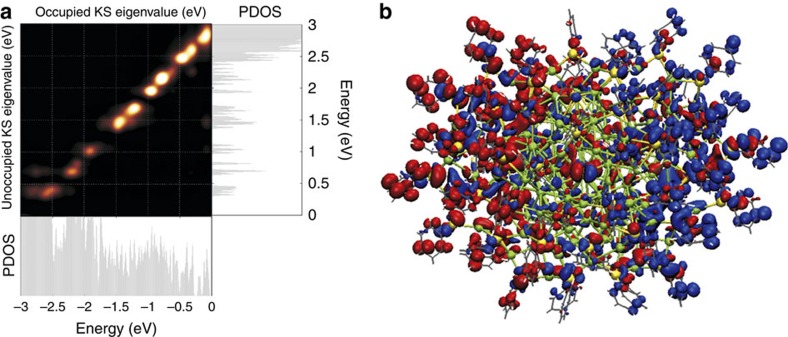
Analysis of the peak at 425 nm (2.9 eV) in the time-dependent DFT (TDDFT) optical spectrum of Ag_136_. (**a**) The transition contribution map shown on the top left reveals that this peak consists of a large number of single-electron particle-hole transitions. Holes are created for states from the Fermi level down to about −2.5 eV (horizontal projected densities of electron states (PDOS) plot) and particles are created for states from the lowest unoccupied molecular orbital (LUMO) state up to ∼2.9 eV (vertical PDOS plot). In that energy range, both silver core states and ligand (sulfur and the pi-system of the phenyl) states are active and the total transition is a mixture of Ag(5 s) to ligand, ligand to Ag(5 s), ligand to ligand and Ag(4d) to Ag(5 s) contributions. (**b**) The induced transition density shows the collective dipole oscillation. Blue and red colours indicate mean deficit and surplus of electron density, respectively.

The optical absorption of the Ag_136_ cluster was studied by using linear-response (LR) time-dependent DFT calculations. The optical spectrum of the atomistic model for Ag_136_ agrees very well with the experimental data, having an overall similar shape in the ultraviolet–visible region and featuring a broad peak at 425 nm (2.9 eV; Fig. 5a and Supplementary Fig. 21). Analysis of this peak (Fig. 6) shows that it is composed of several contributions including Ag(5*s*) to ligand, ligand to Ag(5*s*), ligand to ligand and Ag(4*d*) to Ag(5*s*) transitions. The total transition induced density shows a collective dipole oscillation localized across the interface of the metal core with the ligand layer. We interpret this as a plasmonic feature where the pi-electron density of the electron-rich ligand layer contributes to the collective oscillation. Based on this information, we interpret the experimental peak of Ag_374_ at 465 nm as of similar origin, where the slight red shift compared with the experimental peak of Ag_136_ is attributed to the much larger size of the system.

## Discussion

This report demonstrates that it is feasible to deliberately and reproducibly synthesize metal nanoparticles with specific molecular products that readily enter the truly metallic regime with the emergence of SPR. As miniatures of fivefold twinned nanostructures of fcc metals, the success in resolving the total structures of Ag_136_ and Ag_374_ nanoparticles using single-crystal X-ray diffraction provides important models to visualize at the atomic scale the modest distortion from ideality inside fivefold twinned nanostructures of fcc metals and to describe their detailed surface structures. The Ag nanoparticles reported in this work have the lowest size for silver to display surface plasmonic properties reported to date. These structures serve as real models to understand how silver nanoparticles can span from the molecular to the metallic regime.

The observed plasmonic peaks of Ag_136_ and Ag_374_ are below the threshold energy of the well-known Mie plasmon of 3.5 eV for silver (dipolar bulk limit)^51^. In several gas phase, matrix isolation and surface studies in the past, atomic silver clusters from hundreds of atoms down to a few atoms exhibit resonance absorption at energies that are well over the Mie energy^34^,^52^. The fact that the thiol-stabilized Ag_136_ and Ag_374_ support an even lower bulk limit for the SPR, compared with the classical Mie energy for silver, is a manifestation of the important role played by the interfacial interactions between the thiolate layer and the nanoparticulate metal core. This clearly demonstrates that modifying the interface chemistry by introducing an organic passivating layer that has electronic properties distinct from thiolates could modify the plasmonic behaviour of ligand-stabilized silver nanoparticles. Changes in the interfacial chemistry may provide scope to vary the adjustments in the core and the interfacial structure as well, opening a range of chemical possibilities through which nanoparticle structures and electronic properties can potentially be modified.

## Methods

### Synthesis of the Ag-SPh*t*Bu complex precursor

The complex precursor was prepared by following the reported procedure^53^. In a typical synthesis, 4-*t*BuPhSH (0.38 ml, 2.3 mmol) and NEt_3_ (0.32 ml, 2.3 mmol) were mixed together in 8 ml C_2_H_5_OH. The mixture was then added dropwise to a solution of AgNO_3_ (274 mg, 1.6 mmol) in 5 ml CH_3_CN under stirring. The mixture became a clear yellow solution on stirring overnight at room temperature under a nitrogen atmosphere. The yellow solution was dried under vacuum, to remove solvent yielding a yellow powder of the polymeric Ag-SPh*t*Bu precursor, {(HNEt_3_)_2_[Ag_10_(SPh*t*Bu)_12_]}_*n*_. This was stored in the dark under ambient conditions and used as the precursor for the syntheses of Ag_136_ and Ag_374_ nanoparticles.

### Synthesis and crystallization of Ag_136_ nanoparticles

Thirty mlligrams of the Ag-SPh*t*Bu precursor (containing 92 μmol Ag and 110 μmol SPh*t*Bu^−^ based on the formula of {(HNEt_3_)_2_[Ag_10_(SPh*t*Bu)_12_]}_*n*_) were added to a mixed solvent of dichloromethane and methanol in a volume ratio of 4:1. The solution was cooled to 0 °C in an ice bath and 12 mg PPh_4_Br (29 μmol) was added to the solution. After 5 min stirring, 1 ml NaBH_4_ aqueous solution (45 mg ml^−1^, 1.2 mmol) and 50 μl triethylamine (360 μmol) were added quickly under vigorous stirring. The reaction was aged for 12 h at 0 °C. The aqueous phase was removed and the organic phase was washed several times with water. The solvent was then evaporated to give a dark solid. Black prism-like crystals were crystallized from CH_2_Cl_2_/hexane after 20 days at 4 °C. The synthesis of Ag_136_ with the substitution of Ph_4_PBr by an equivalent of Ph_4_PCl also results in Ag_136_.

### Synthesis and crystallization of Ag_374_ nanoparticles

Thirty milligrams of the Ag-SPh*t*Bu precursor were added to a mixed solvent of dichloromethane and methanol. Twelve milligrams of AgBF_4_ (62 μmol) and 12 mg PPh_4_Br (29 μmol) were added sequentially. After 5 min stirring, 1 ml NaBH_4_ aqueous solution (45 mg ml^−1^, 1.2 mmol) and 50 μl triethylamine (360 μmol) were added quickly under vigorous stirring. The reaction was aged for 4 h at room temperature. The aqueous phase was discarded and the mixture in organic phase was washed several times with water and evaporated to give a dark solid. Black block-like crystals were crystallized from CH_2_Cl_2_/hexane after 2 months at 4 °C.

### X-ray single-crystal analysis

Diffraction data of the single crystals grown from the solutions of Ag_136_ and Ag_374_ nanoparticles were collected on an Agilent Technologies SuperNova system X-ray single-crystal diffractometer with Cu K*α* radiation (*λ*=1.54184 Å) at 100 K to a resolution of 1.2 Å. The data were reduced using CrysAlis^Pro^. The structures were solved in ShelXL and refined using a combination of ShelXT, Olex2, Shelxle and CRYSTALS^37^,^38^,^39^,^40^,^41^,^42^. A customized refinement procedure employing techniques from both chemical crystallographic practice and macromolecular modelling methods (including contoured electron density mapping of all *t*BuPh groups) was evolved to address the challenges of each structure. In Ag_136_, a silver site of less than half occupancy has been modelled, which may be occupied in some molecules. The associated electron density refines well on this basis and the assignment makes chemical sense, noting that a sulfur atom with a high displacement parameter lies closer to this site than is reasonable. It is proposed that when this additional silver is present, the close sulfur atom lies in a site at the end of the sulfur ellipsoid furthest from the silver site. Significant ‘solvent voids' are noted and it is proposed that these regions are more properly termed ‘frozen solution' than solvent of crystallization in view of the very limited localized electron density features lying in the voids (note CH_2_Cl_2_ is the main solvent). The modest observed residual electron densities lie within the silver nanoparticles. No modifications to the data or refinement were made to account for these void volumes (see Supplementary Discussion and Supplementary Methods for further crystallographic details).

### Computational method

The electronic structures of Ag_136_ and Ag_374_ were studied by DFT code GPAW, which implements projector-augmented wave method in a real-space grid^54^. The real space had a grid spacing of 0.2 A°. Ag(4*d*^10^5*s*^1^), S(3*s*^2^3*p*^4^), Br(4*s*^2^4*p*^5^) and H(1*s*^1^) electrons were regarded as the valence, and the projector-augmented wave setups for Ag included scalar-relativistic corrections. Structures and total energies were evaluated at the gradient-corrected functional of Perdew, Burke and Ernzerhof (PBE) level^55^. It should be pointed out that the configurations for Ag_136_ and Ag_374_ used in the calculations were taken from our preliminary crystallographic model. This Ag_136_ model had a formula of [Ag_136_(SPh*t*Bu)_65_Br_2_]^−^ which had 70 metallic free electrons (from the superatom counting rules). The electron number was same as that for the dominant final refined structure [Ag_136_(SPh)_64_Cl_3_]^−^. The correction to the structural formula for Ag_136_ does not affect our conclusions concerning the electronic structure and optical properties. The preliminary crystallographic model for Ag_374_ had a formula of [Ag_374_(SR)_115_Br_2_] containing two more SR^−^ groups than that of the final refined structure again. These two sites were ultimately modelled with Cl at the former S site, yielding the same electron count. The organic groups were replaced by SPh for Ag_136_, after which the organic layer was relaxed to an energy minimum, while keeping the Ag, S and Br atoms fixed to experimental positions. For Ag_374_, a simplified ligand SR=SH was used and the S–H bonds were relaxed, while keeping Ag, S and Br atoms fixed to their experimental positions. Angular momentum analysis of the Kohn–Sham orbitals was done as metal-core projected density of states as described earlier^56^.

The optical absorption spectrum of Ag_136_ was calculated with the PBE level using time-dependent DFT formalism in GPAW.^57^ The grid spacing was 0.8 Å. The electron density and wave functions were calculated by the local density approximation and the PBE functional was used for calculating LR optical absorption spectra. The transitions contributing to selected optical peaks were analysed using a recently developed method based on time-dependent density functional perturbation theory^58^.

### Data availability

The X-ray crystallographic coordinates for structures reported in this article have been deposited at the Cambridge Crystallographic Data Centre, under deposition number CCDC-1496141 (Ag_374_) and 1496142 (Ag_136_). These data can be obtained free of charge from the Cambridge Crystallographic Data Centre via www.ccdc.cam.ac.uk/data_request/cif. All other data are available from the authors on reasonable request.

## Additional information

**How to cite this article:** Yang, H. *et al*. Plasmonic twinned silver nanoparticles with molecular precision. *Nat. Commun.* 7:12809 doi: 10.1038/ncomms12809 (2016).

## Supplementary Material

Supplementary Information Supplementary Figures 1 - 21, Supplementary Tables 1 and 2, Supplementary Discussion, Supplementary Methods and Supplementary References

Supplementary Data 1 Crystallographic information file for Ag_374_.

Supplementary Data 2 Crystallographic information file for Ag_374_.
