# Supplementary material for: Plasmonic twinned silver nanoparticles with molecular precision
Source: Nat Commun. 2016 Sep 9;7:12809. doi: 10.1038/ncomms12809 (PMC5023969; doi:10.1038/ncomms12809)
Supplement: Supplementary Information — Supplementary Figures 1 - 21, Supplementary Tables 1 and 2, Supplementary Discussion, Supplementary Methods and Supplementary References [file ncomms12809-s1.pdf]

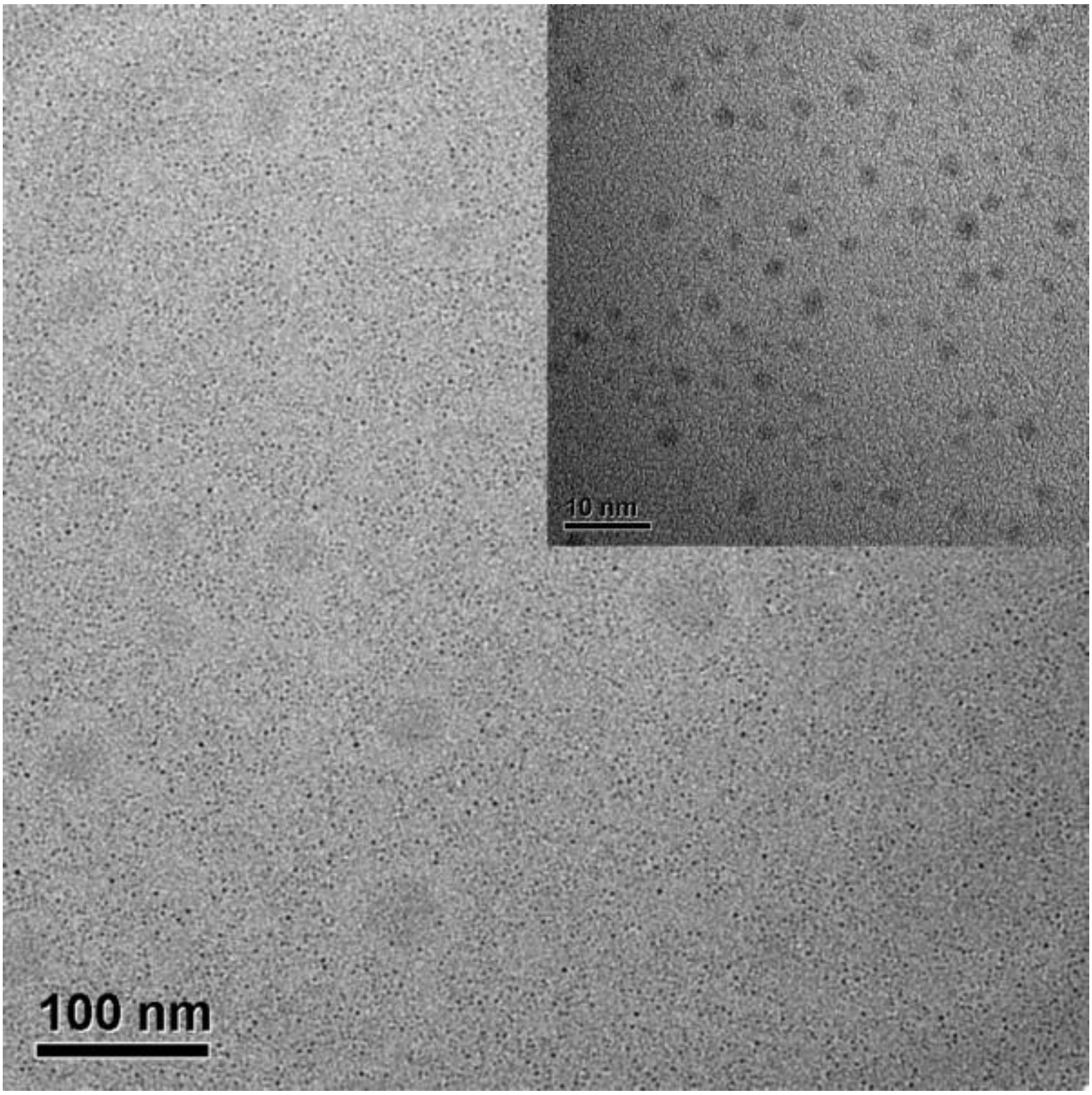

Supplementary Figure 1. TEM images of the as-prepared small Ag_{136} nanoparticles. The nanoparticles are stabilized by 4-*tert*-butylbenzenethiolate. The inset is a representative high-magnification TEM image of the nanoparticles.

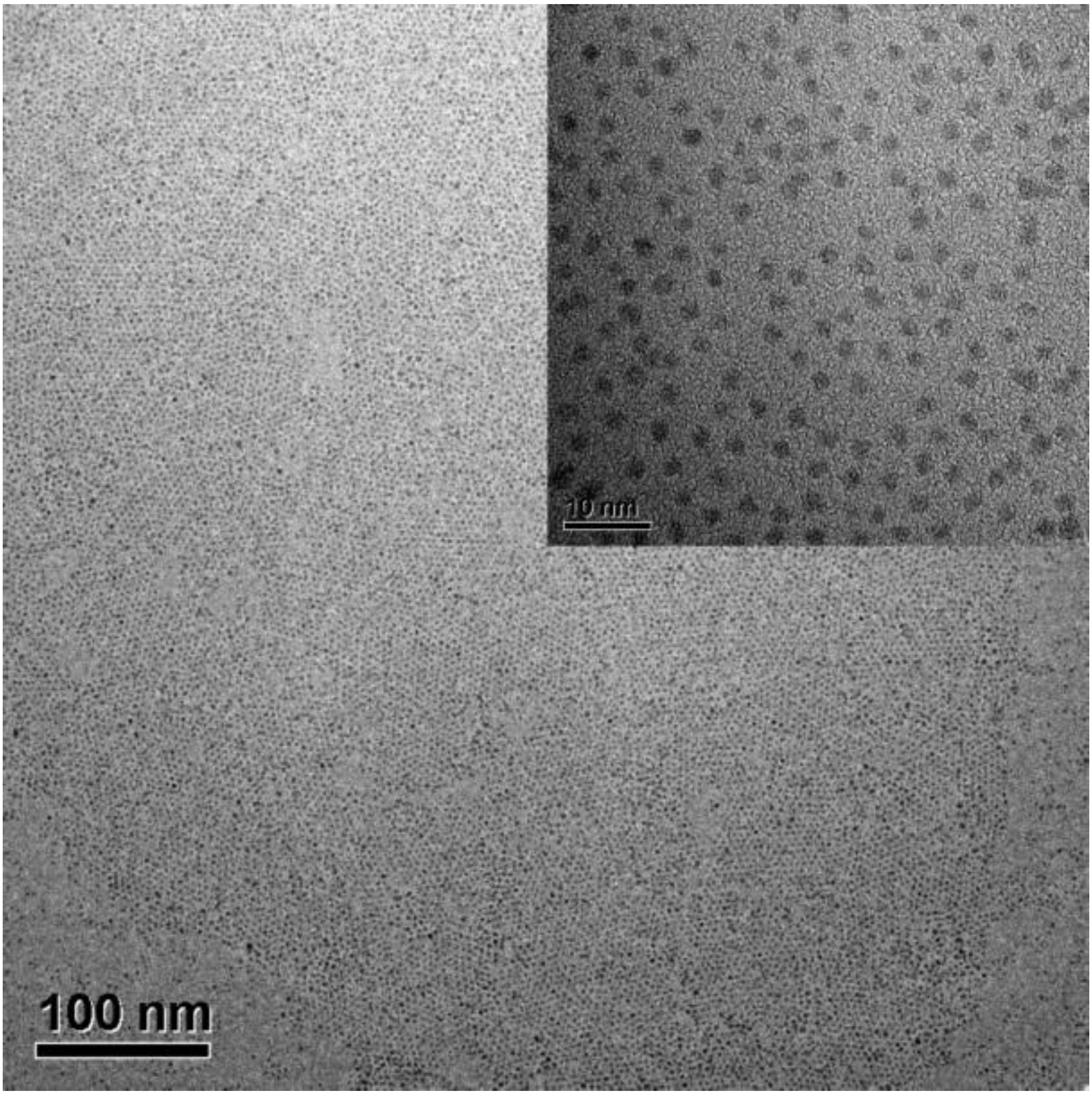

Supplementary Figure 2. TEM images of the as-prepared small Ag_{374} nanoparticles. The nanoparticles are stabilized by 4-*tert*-butylbenzenethiolate. The inset is a representative high-magnification TEM image of the nanoparticles.

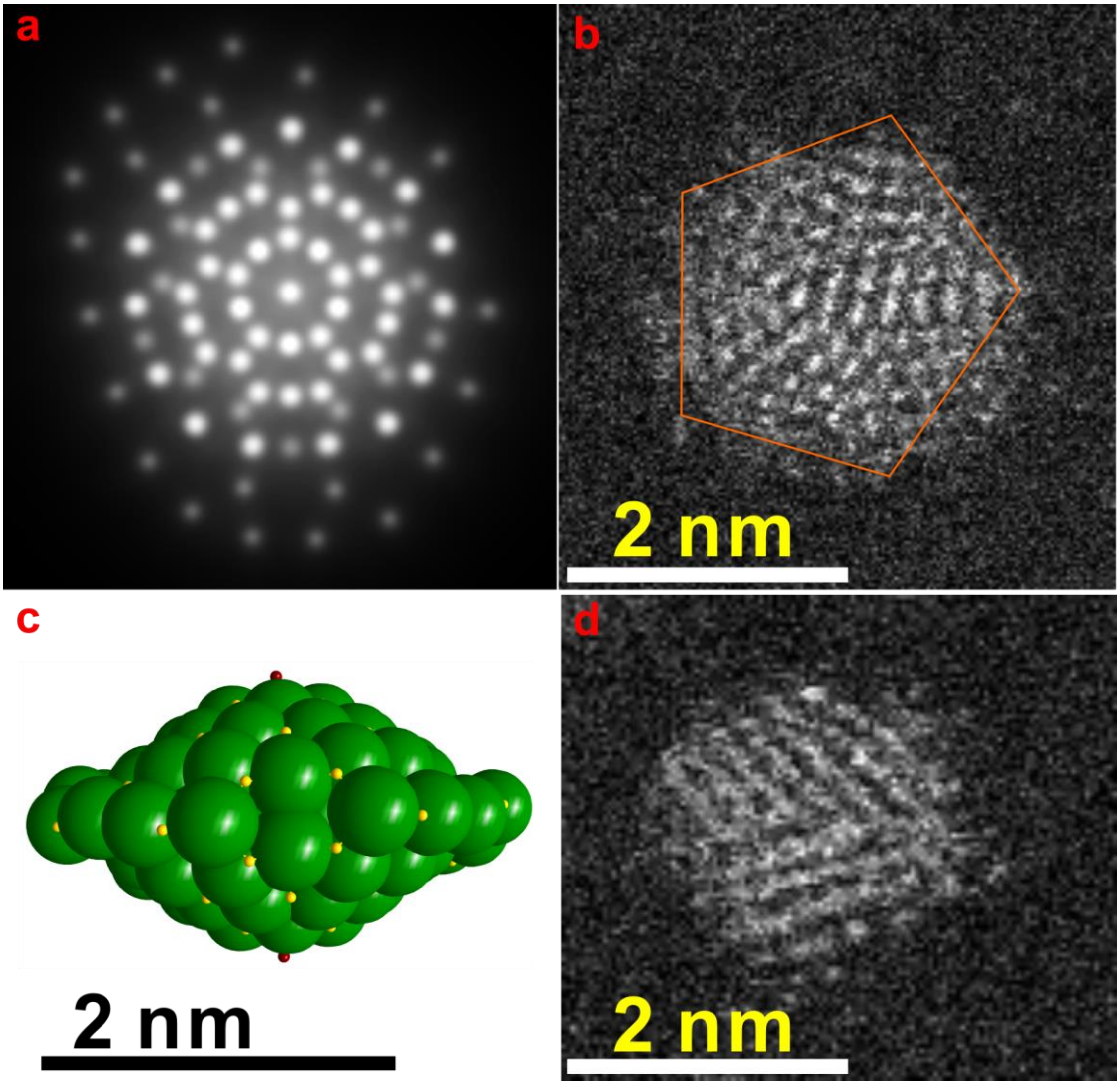

Supplementary Figure 3. High-resolution TEM images of Ag_{136} nanoparticle. Top-view (a) simulated and (b) experimental HRTEM images of the nanoparticle; (c) Side-view space-filling model and (d) experimental HRTEM image of the nanoparticle.

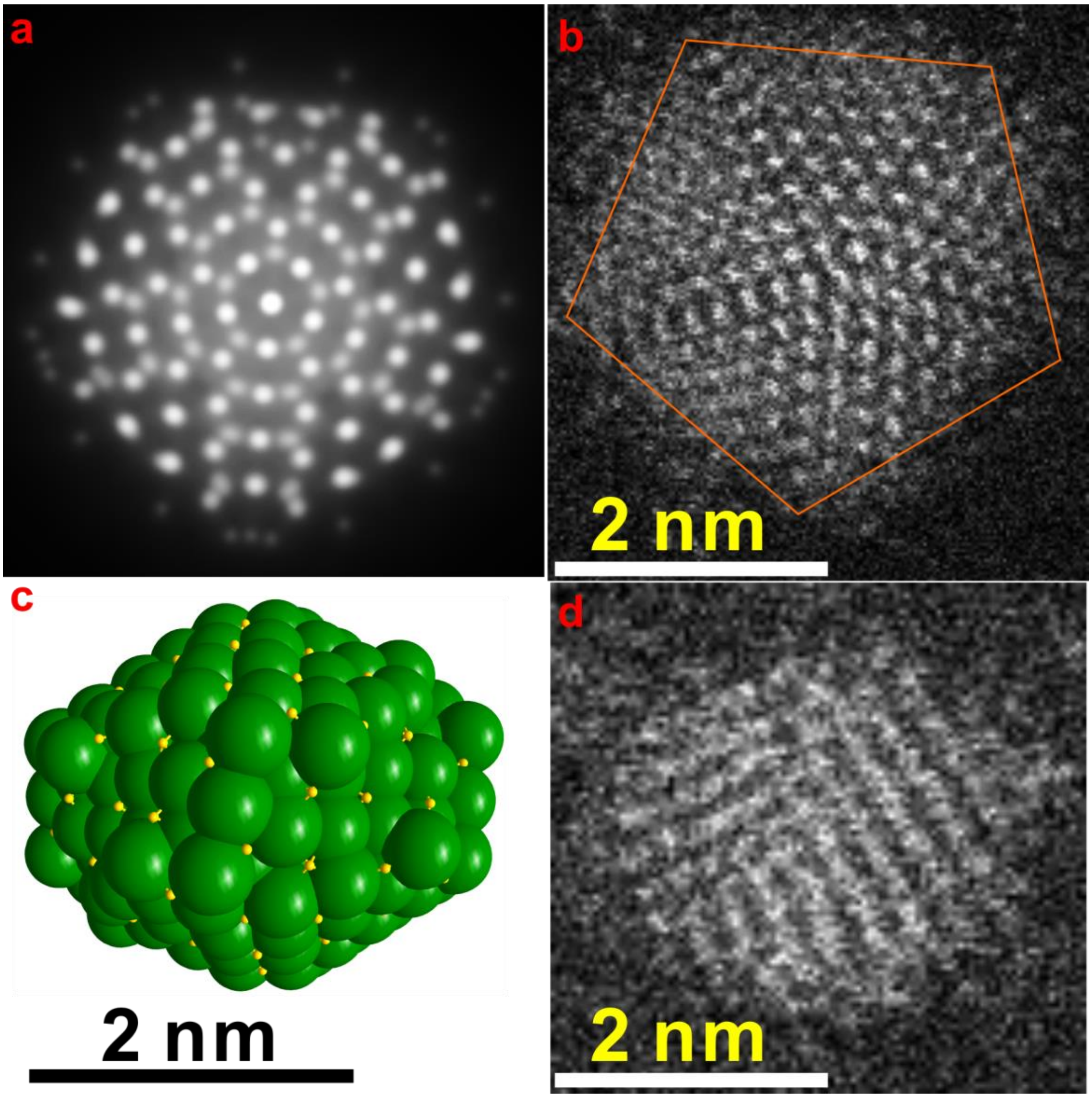

Supplementary Figure 4. High-resolution TEM images of Ag_{374} nanoparticle. Top-view (a) simulated and (b) experimental HRTEM images of the nanoparticle; (c) Side-view space-filling model and (d) experimental HRTEM image of the nanoparticle.

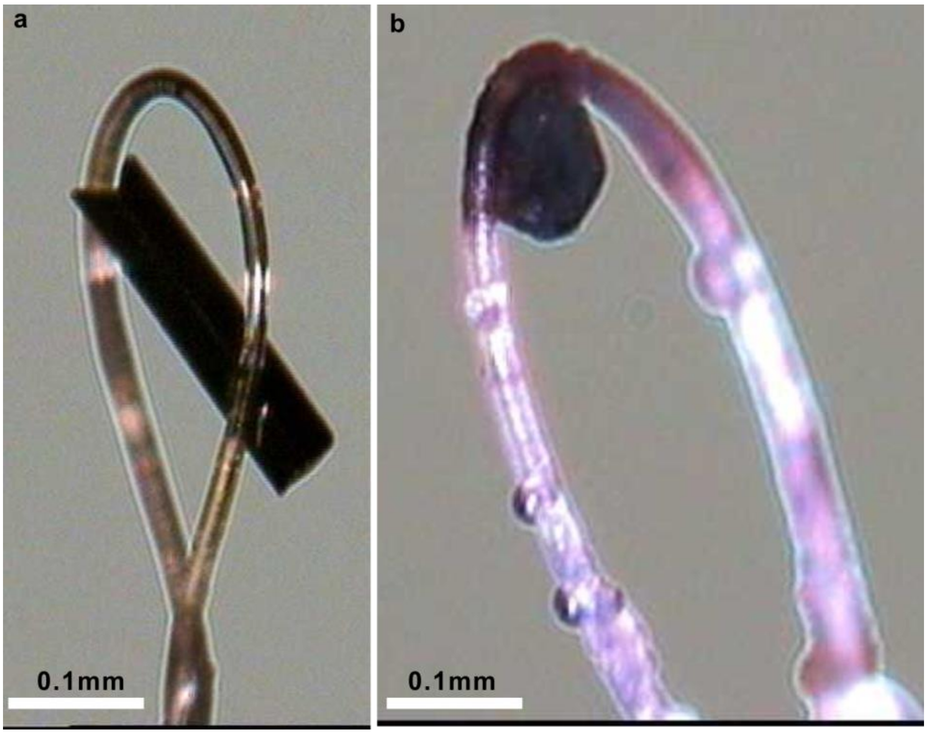

Supplementary Figure 5. Photographs of single crystals of Ag nanoparticles. These high-quality single crystals of (a) Ag_{136} and (b) Ag_{374} nanoparticles were suitable for X-ray single crystal analysis.

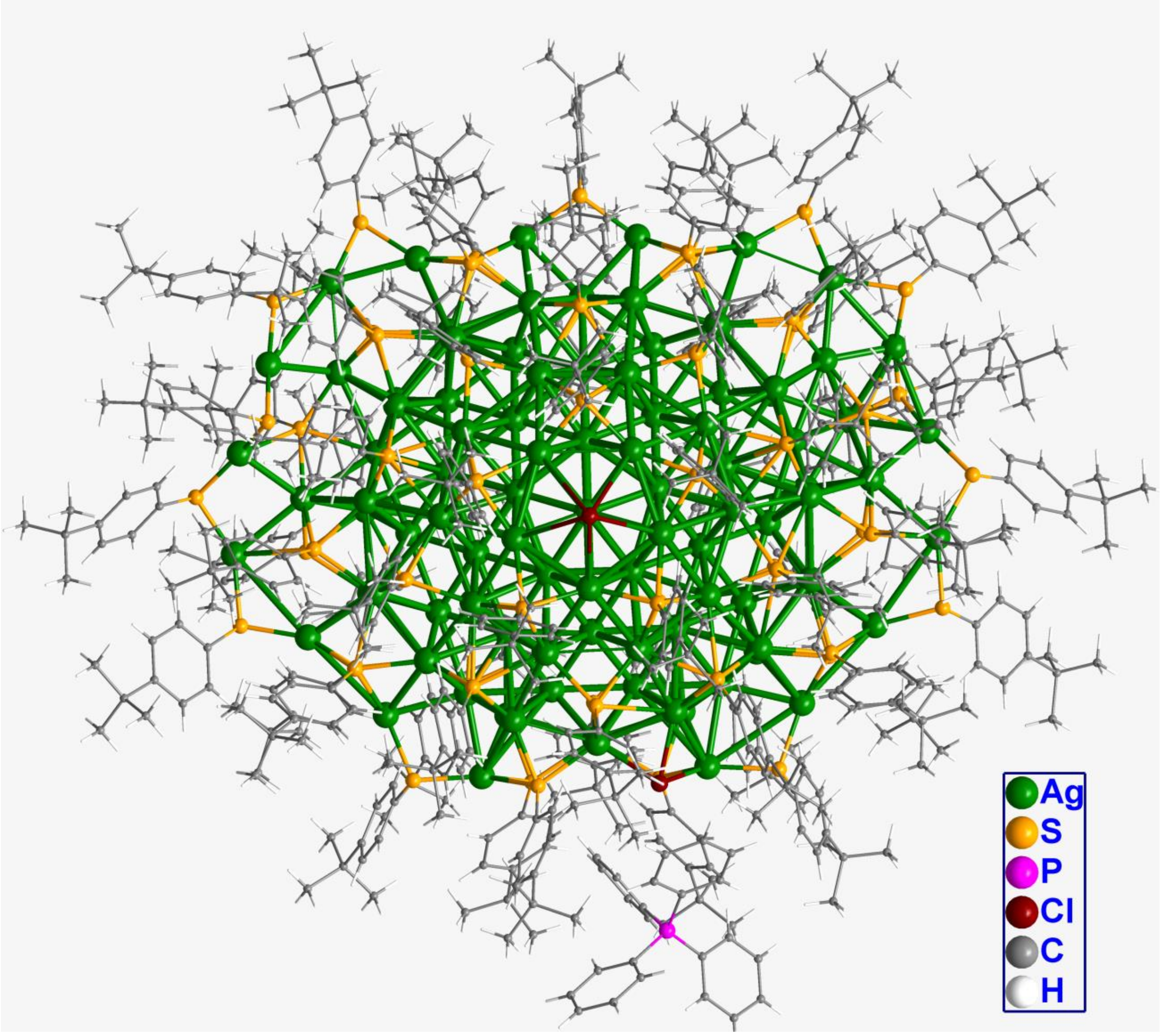

Supplementary Figure 6. The crystallographically determined structure of the ion pair of $[Ag_{136}(SR)_{64}Cl_3]^-$ and PPh_4^+. Color legend: green sphere, Ag; yellow sphere, S; pink sphere, P; brown sphere, Cl; grey sphere, C; white sphere, H.

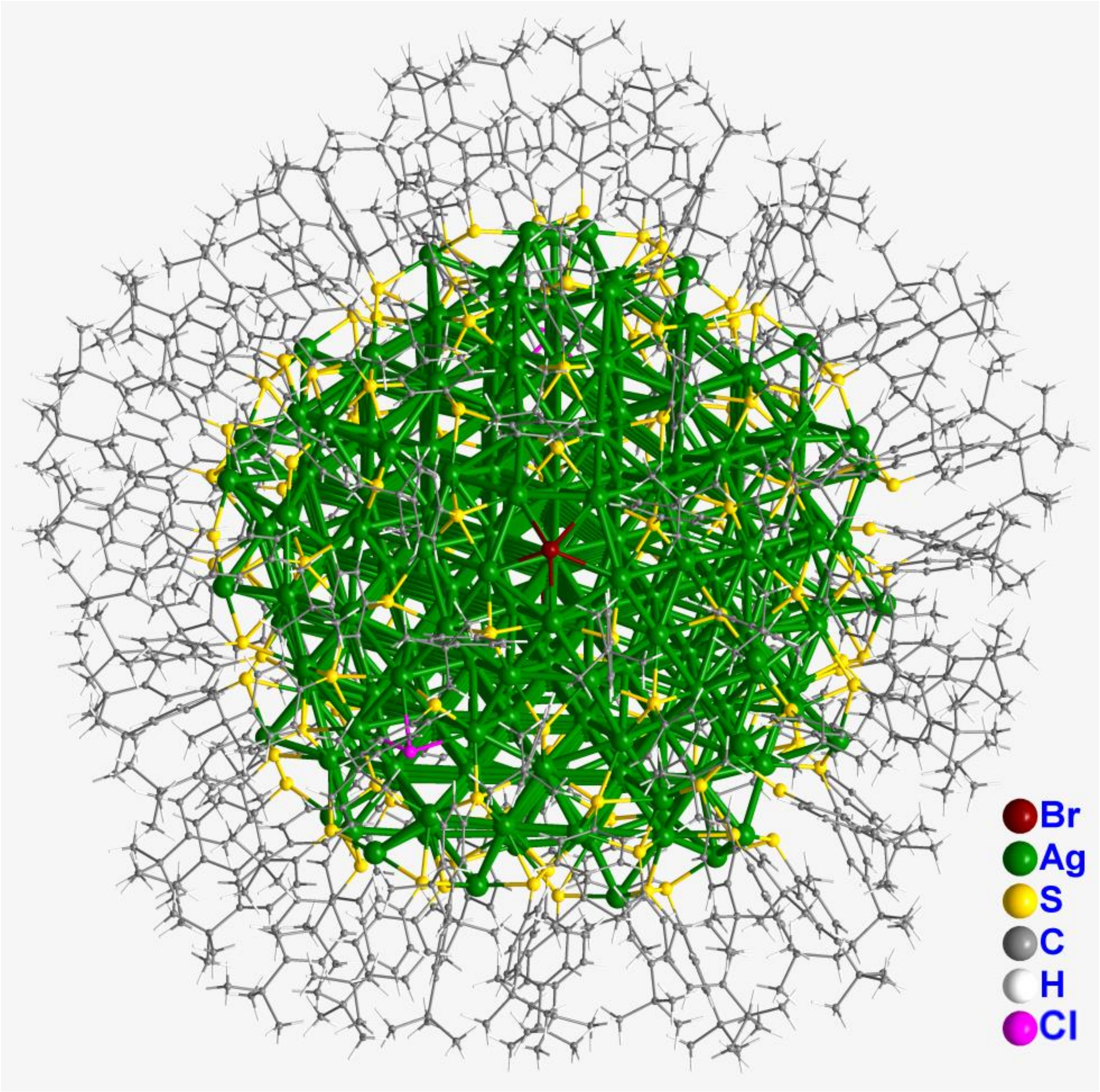

Supplementary Figure 7. The crystallographically determined structure of $[Ag_{374}(SR)_{113}Br_2Cl_2]$. Color legend: green sphere, Ag; yellow sphere, S; pink sphere, Cl; brown sphere, Br; grey sphere, C; white sphere, H.

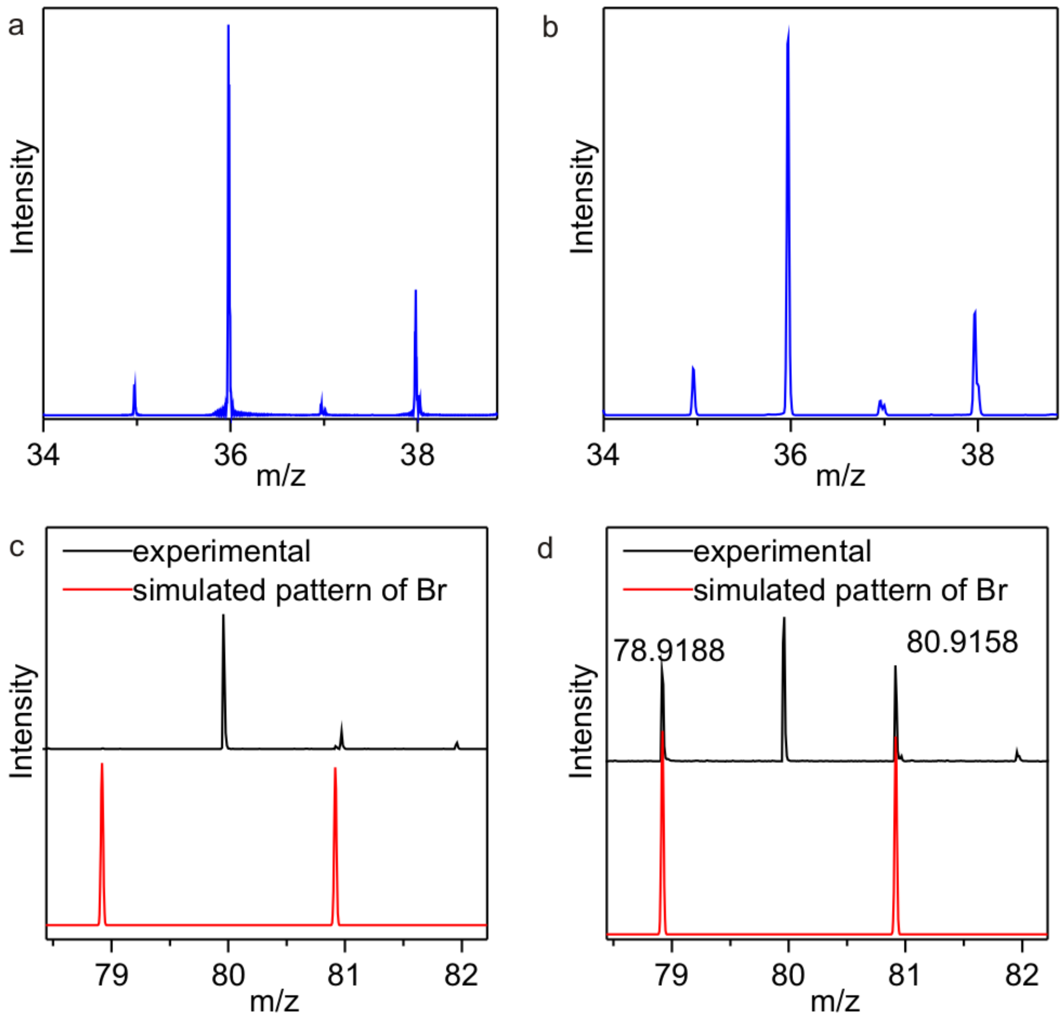

Supplementary Figure 8. TPD-MS spectra of Ag136. (a) TPD-MS spectrum corresponding to liberated Cl/HCl species from Ag_{136}. (b) TPD-MS spectrum corresponding to liberated Cl/HCl species from [Ag_{374}. (c) ESI-MS spectrum of Ag_{136} showing the absence of Br. (d) ESI-MS spectrum of Ag_{374} showing the presence of Br. The TPD-MS experiment was performed on a home-made TPD-TOF analyzer. Samples were pyrolyzed in a small tube heated by a heating coil. A K-type thermocouple was put inside the sample tube and insulated from samples to measure the temperature. The heating coil was powered by a precise electric source and adjusted at interval of 10mV. The temperature of sample tube was ramped from room temperature to 400°C smoothly at a rate of 5 K/min controlled by computer. The desorbed spices were ionized by an electron beam ion source (EBIS), and then transferred to the TOF analyzer by an ion optical system. All steps proceeded at high vacuum of about 3×10^{-5} Pa. The mass spectrum and sample temperature were acquired and recorded every second. Each spectrum is an accumulation of 10000 spectra gathered at interval of 100 μs. ESI-MS was performed using THF solutions of clusters after ultrasonic treatment.

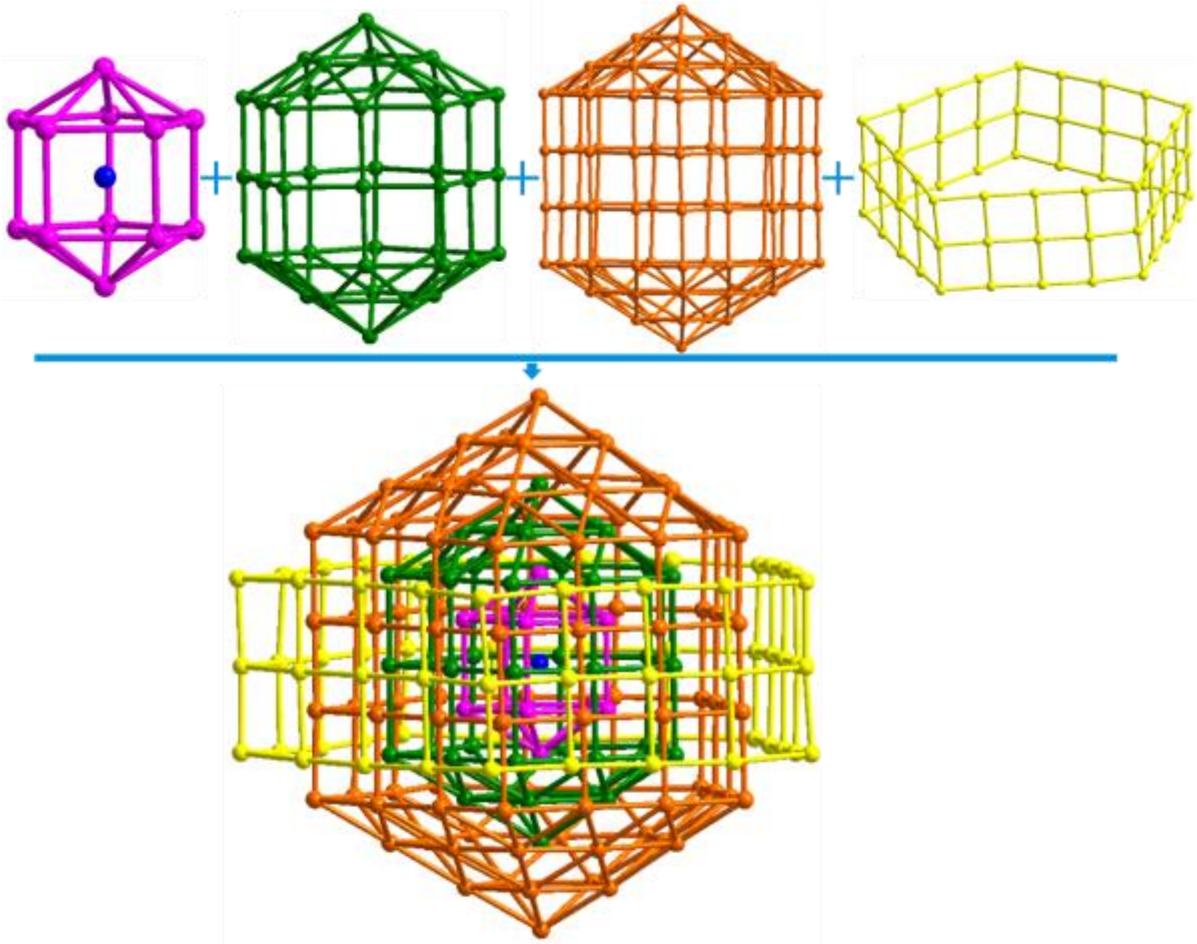

Supplementary Figure 9. The Ag_{207} core structure of [$Ag_{374}(SR)_{113}Br_2Cl_2$]. The core is formed by one central Ag atom (blue sphere) successively surrounded by three closed shells consisting of 12 (pink), 42 (green) and 92(orange) atoms, and a pentagonal-cylinder layer of 60 Ag atoms (yellow) about the five-fold axis. Each closed shell is an elongated pentagonal bipyramid (J16 Johnson solid).

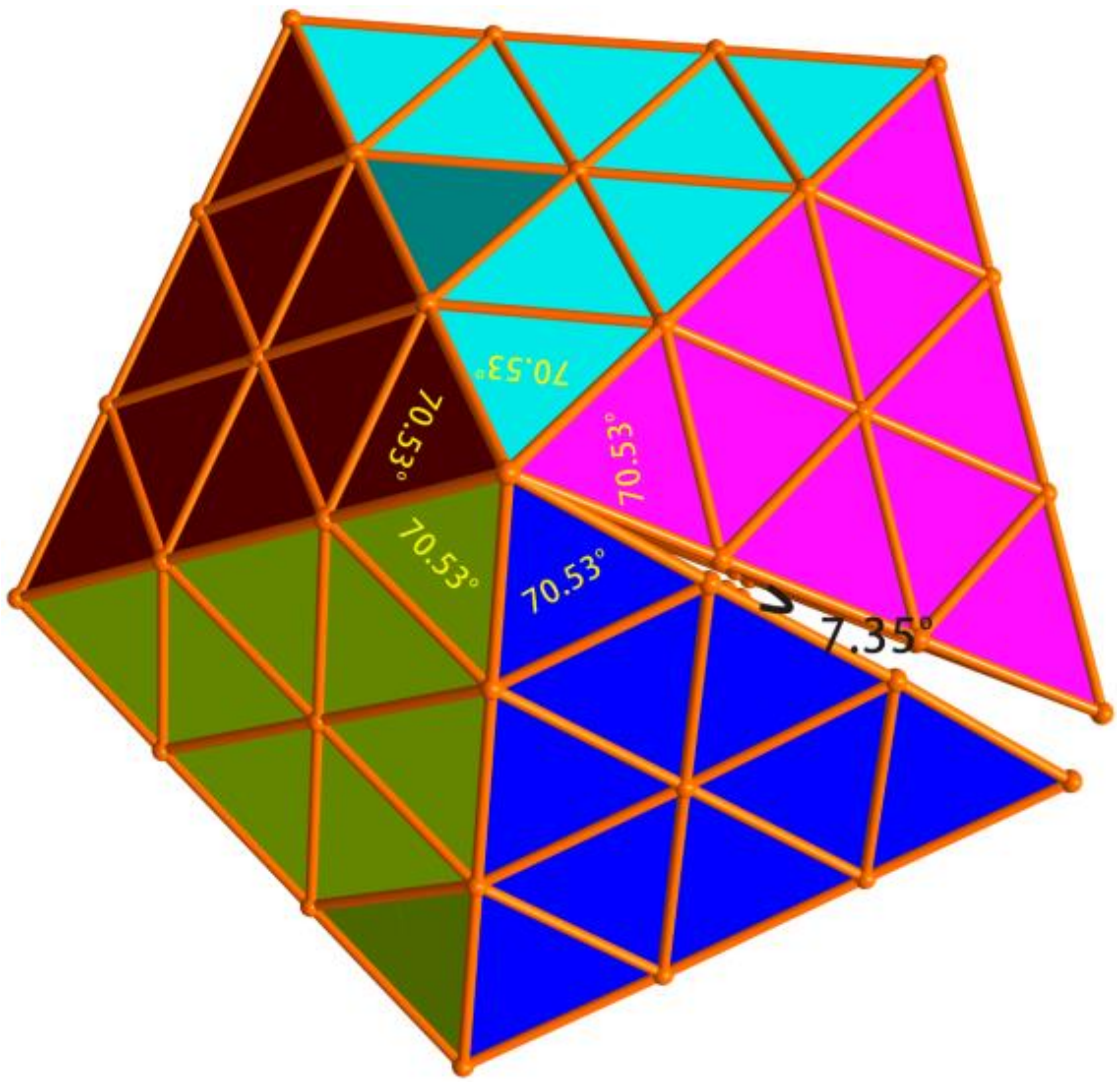

Supplementary Figure 10. Decahedral (pentagonal bipyramidal) cluster constructed from five regular tetrahedral subunits. For uniform perfect f.c.c. tetrahedral subunits, the angle between adjacent (111) faces, illustrated here in the [110] projection, is 70.53°. As a result, a solid-angle deficiency of 7.35° results from fusion of five such ideal tetrahedral domains.

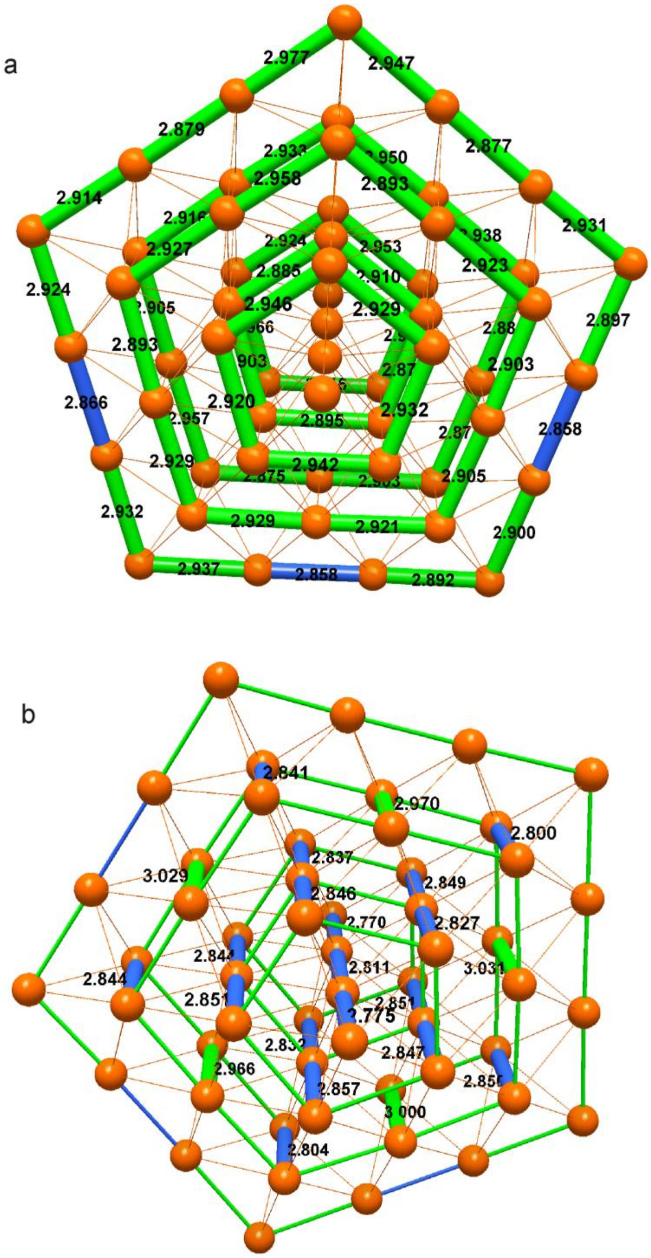

Supplementary Figure 11. The Ag-Ag distance analysis of Ag_{54} core of $[Ag_{136}(SR)_{64}Cl_3]^-$. Major Ag-Ag bonds (18 out of 23) along the five-fold axis are shorter than the average Ag-Ag bond distance (2.870 Å) (a), most Ag-Ag bonds (47 out of 50) perpendicular to the five-fold axis are longer than 2.870 Å (b). Color legend: orange spheres, Ag; green bonds, Ag-Ag bonds longer than 2.870 Å; blue bonds, Ag-Ag bonds shorter than 2.870 Å.

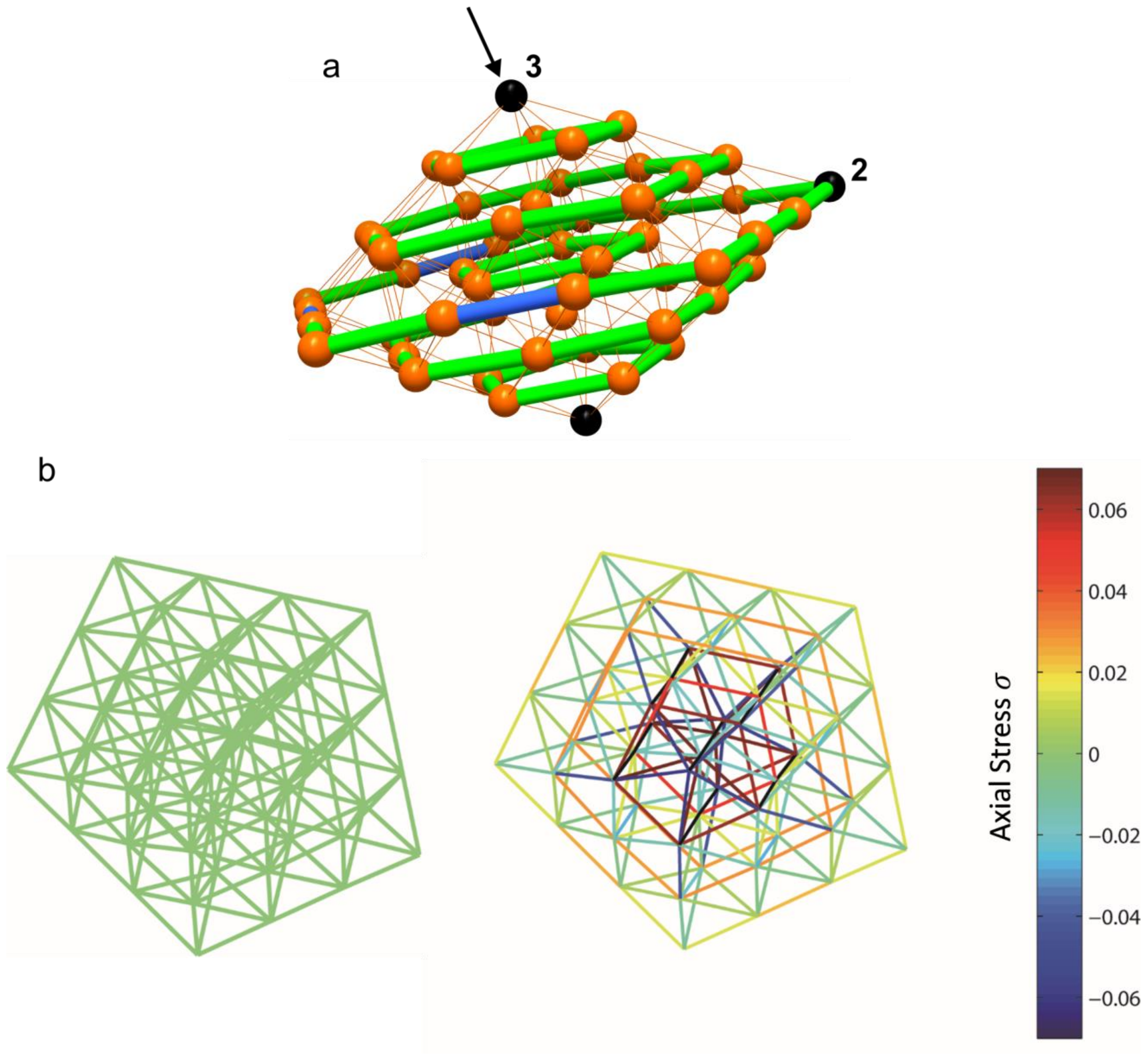

Supplementary Figure 12. The distortion from ideality of the Ag_{54} five-fold twinned decahedron of $[Ag_{136}(SR)_{64}Cl_3]^-$. (a) The Ag_{54} decahedron was taken as a truss structure to do the stress distribution analysis. During the stress distribution analysis, a load P was applied along the five-fold axis and the essential boundary conditions were imposed at nodes 1, 2 and 3 (the black spheres). Node 1 was fixed along all three directions. For node 2, the displacement perpendicular to the plane through 123 was constrained. While node 3 was only allowed to move along line 13. (b) The lattice stress distribution of the truss structure (Ag_{54} five-fold twinned decahedron) with the hypothesis that Young's modulus, cross sectional area of the truss structure, and the load P were all set to be unity. At left, the undeformed structure and at right, the deformed structure. The different color ranging from red to blue indicates different deviation level for each bond (positive value means tension and negative value denotes compression).

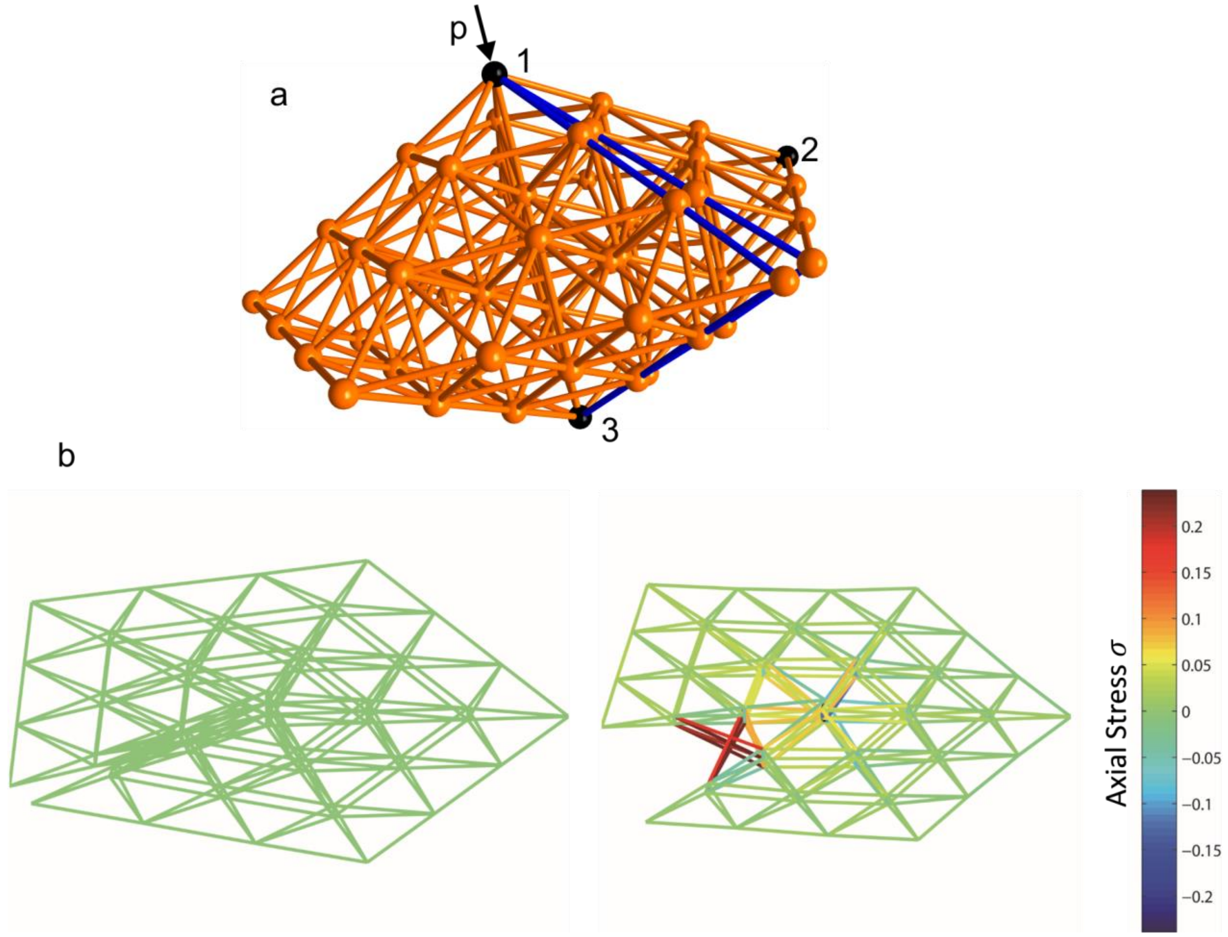

Supplementary Figure 13. The distortion distribution in the decahedron formed by five tetrahedral subunits with solid-angle deficiency. (a) The decahedron was taken as a truss structure to do the stress distribution analysis. During the stress distribution analysis, a load P was applied along the five-fold axis. And the essential boundary conditions were imposed at nodes 1, 2 and 3 (the black spheres). Node 1 was fixed along all three directions. For node 2, the displacement perpendicular to the plane through 123 was constrained. While node 3 was only allowed to move along line 13. (b) The stress distribution of the truss structure (Ag_{54} five-fold twinned decahedron) with the hypothesis that Young's modulus, cross sectional area of the truss structure, and the load P were all set to be unity. The left was the undeformed structure and the right was the deformed structure. The different color ranging from red to blue indicates different strain level for each bond (the positive value means tension and negative value denotes compression).

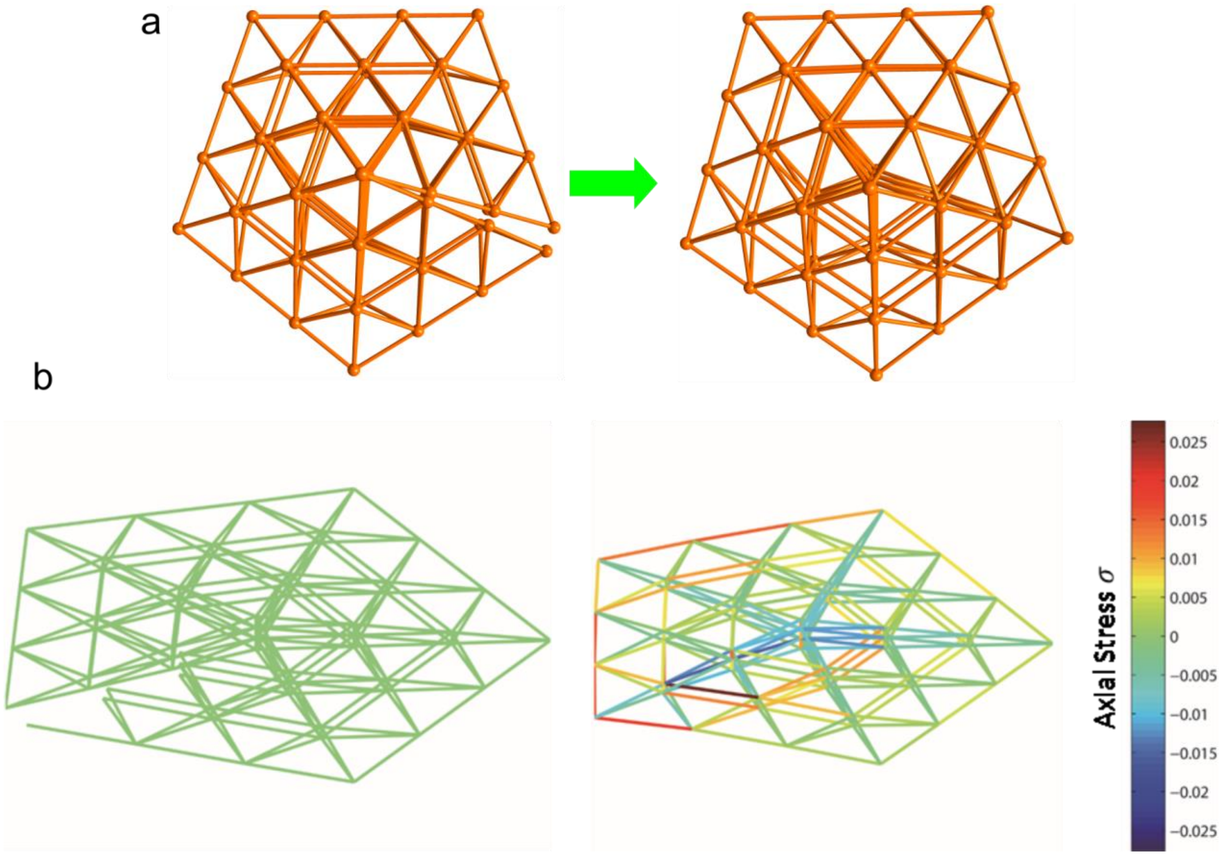

Supplementary Figure 14. The structure distortion distribution for the decahedral mode formed by five tetrahedral subunits with solid-angle deficiency and the decahedron with solid-angle deficiency. It has the same essential boundary condition as in Supplementary Figure 12 and Supplementary Figure 13. The left was the undeformed structure and the right was the deformed structure. The different color ranging from red to blue indicated different stress level for each bond (the positive value means tension and negative value denotes compression).

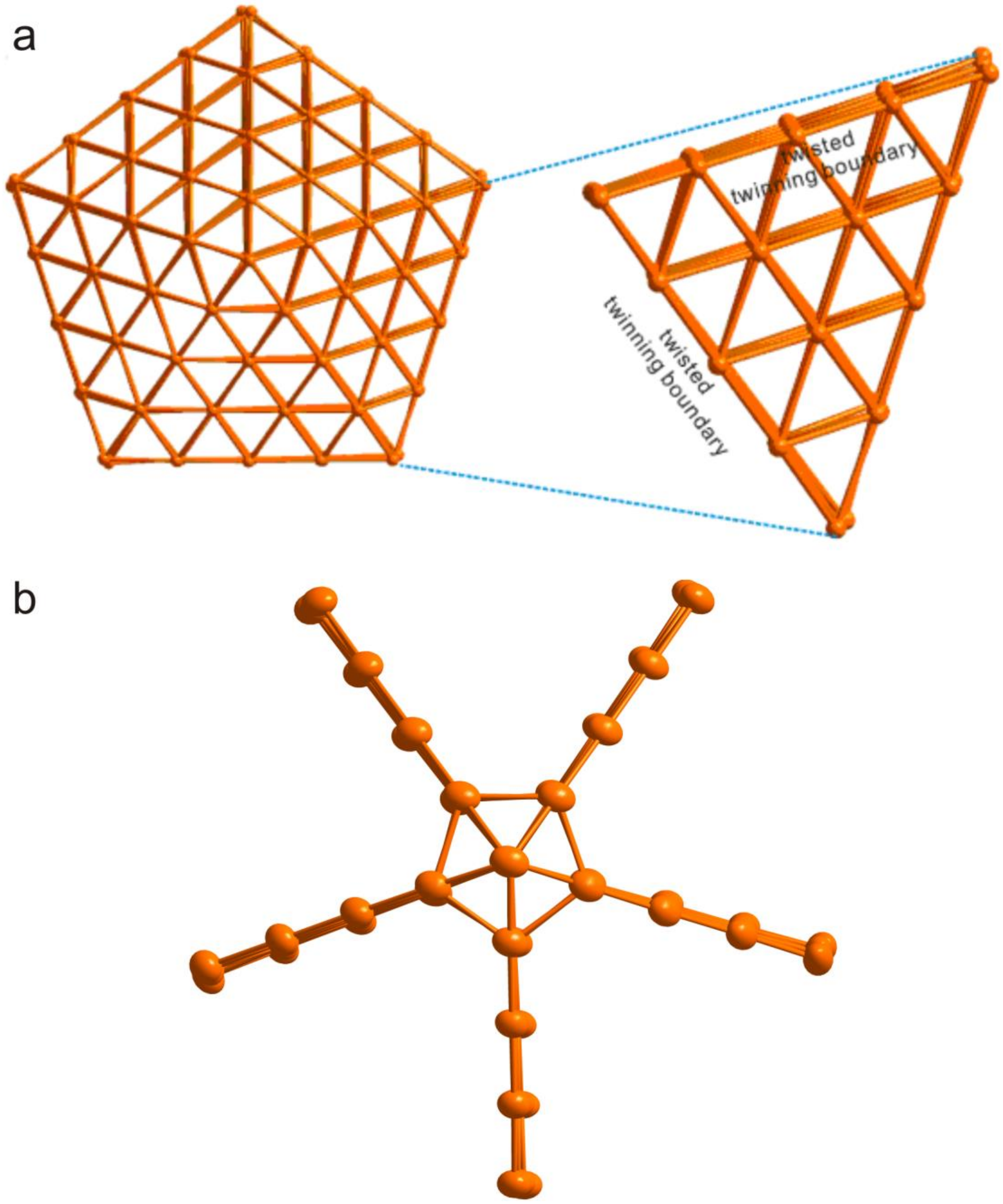

Supplementary Figure 15. The five-fold twin boundary (111) faces in the Ag_{207} core are not strictly planar. (a) The diagram showing the twisted twinning boundaries. (b) The twin boundary sites viewed from central atom perpendicular to the molecular equatorial plane projected toward the apex. It is noted that even the atoms of the (J73)/2 cap deviate little from these twin bounding planes.

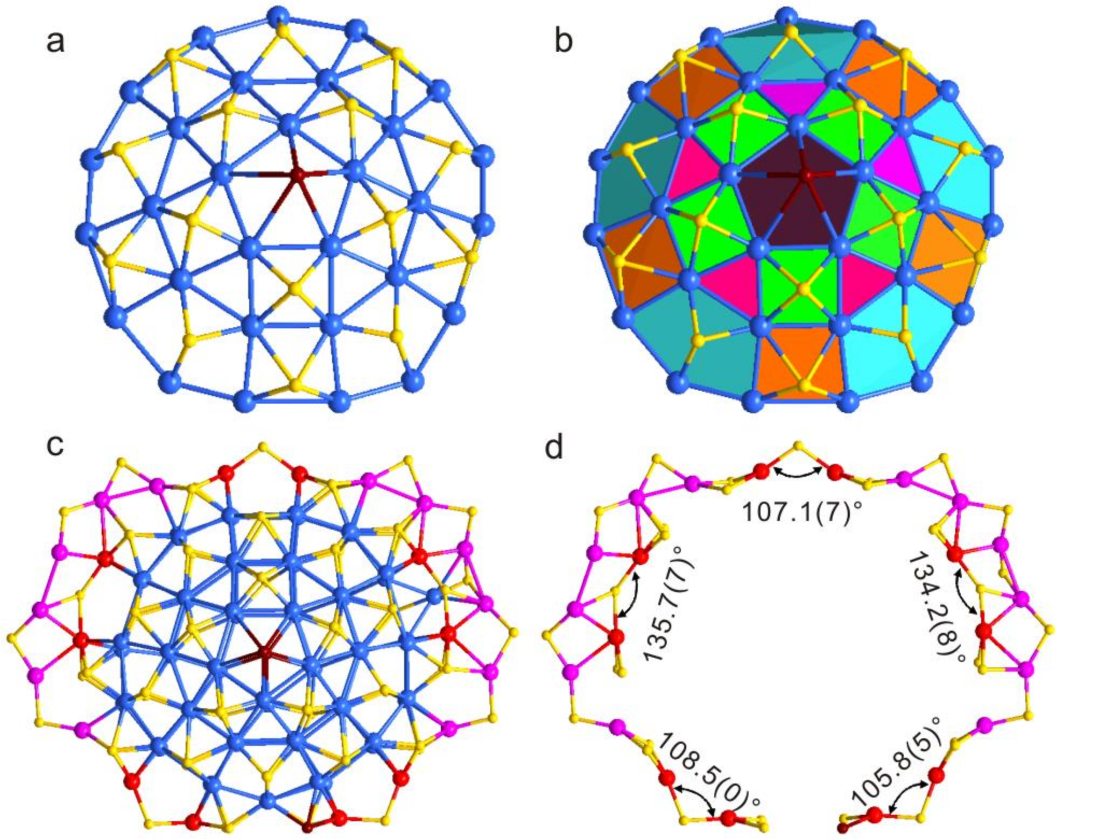

Supplementary Figure 16. The Ag-SR complex outer shell of $[Ag_{136}(SR)_{64}Cl_3]^-$. (a, b) The bowl-shape $[Ag_{30}(SR)_{15}Cl]$ capping unit; The silver sites describe the vertices of one half of a J73 Johnson solid. The opposite cap is in the eclipsed configuration, thus the two halves do not, correspond to an elongated J73 vertex arrangement. (c) The top-view of whole structure of the$[Ag_{136}(SR)_{64}Cl_3]^-$ complex shell; (d) The equatorial Ag-SR complex shell which links the two half J73 in an eclipsing or mirror image manner. Color legend: blue, red and pink sphere, Ag; yellow sphere, S; brown sphere, Cl.

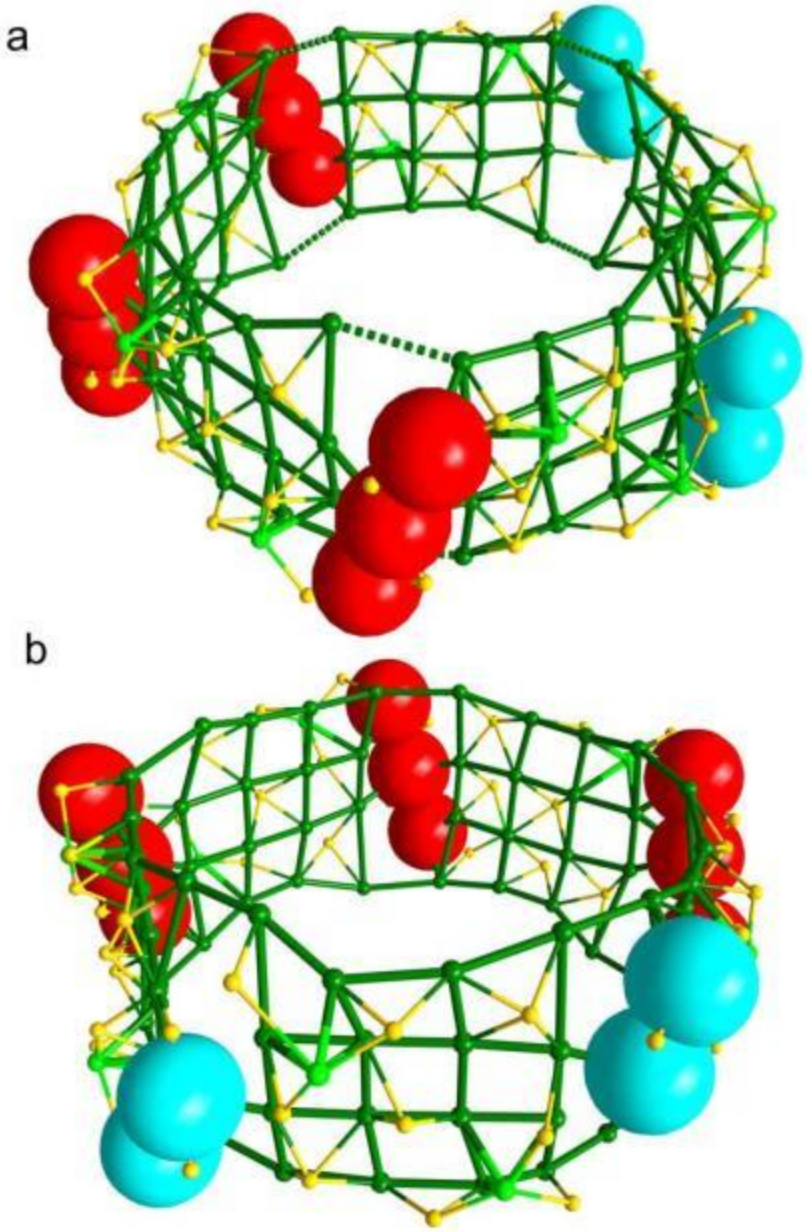

Supplementary Figure 17. The outer drum-like layer of Ag_{374}. In this structure element, five $Ag_{16}(AgS_3)_2S_3$ units are joined together by three $Ag_3(SR)_2$ (red sphere), two $Ag_2(SR)_2$ (blue sphere) motifs and four bridging SR, forming the drum-like layer surrounding the pentagonal prism of the core. Color legend: blue, red and green and light green sphere, Ag; yellow sphere, S.

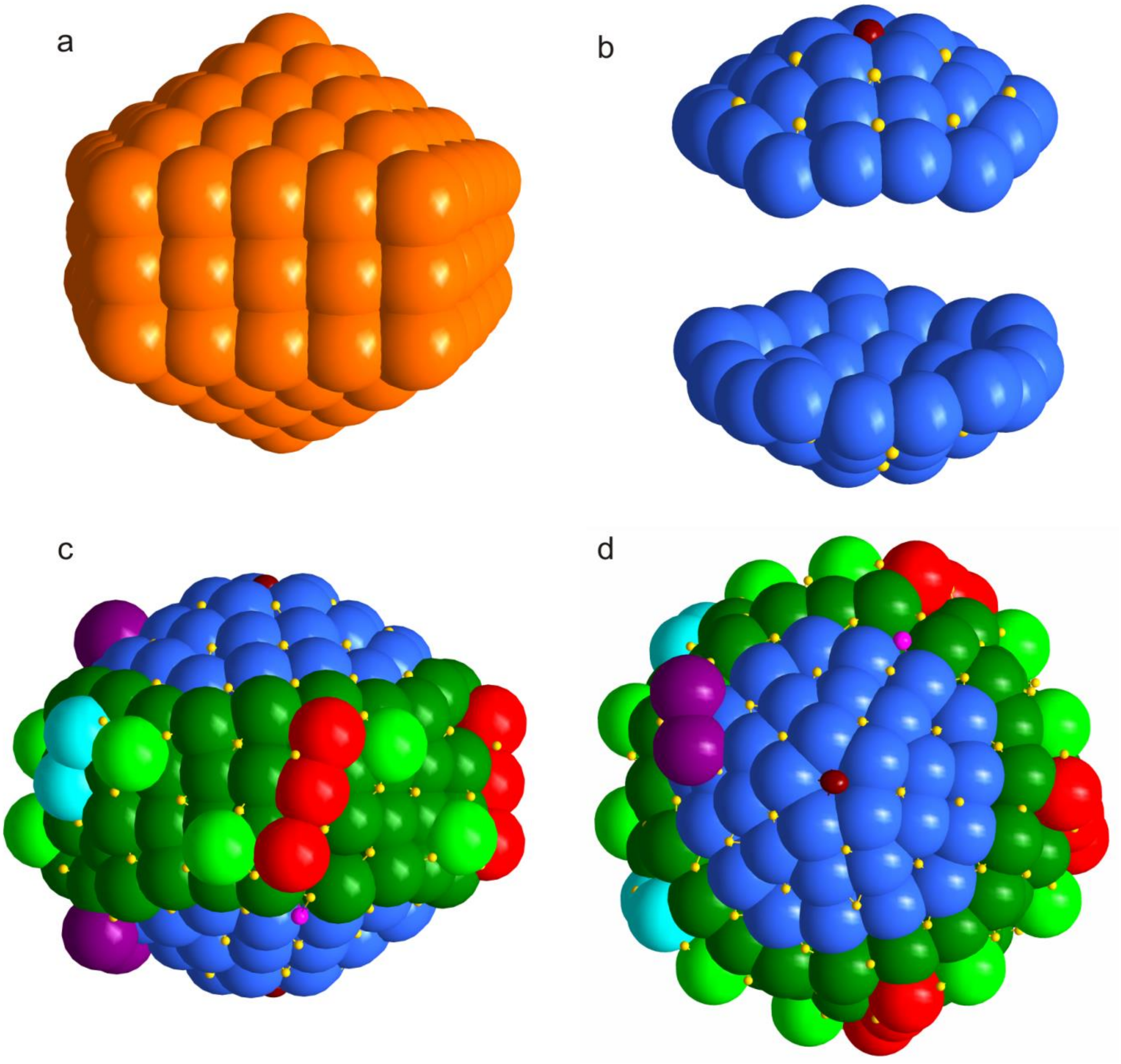

Supplementary Figure 18. The overall structure anatomy of Ag_{374}. (a) The five-fold twinned Ag_{207} core. (b) The bowl-like $[Ag_{30}(SR)_{15}Br]$ capping units at the two ends of Ag_{207}. (c) The side-view and top-view of the outer closed complex shell. Color legend for (b-d): orange, blue, red, green, purple, light green and cyan sphere, Ag; yellow sphere, S; pink sphere, Cl.

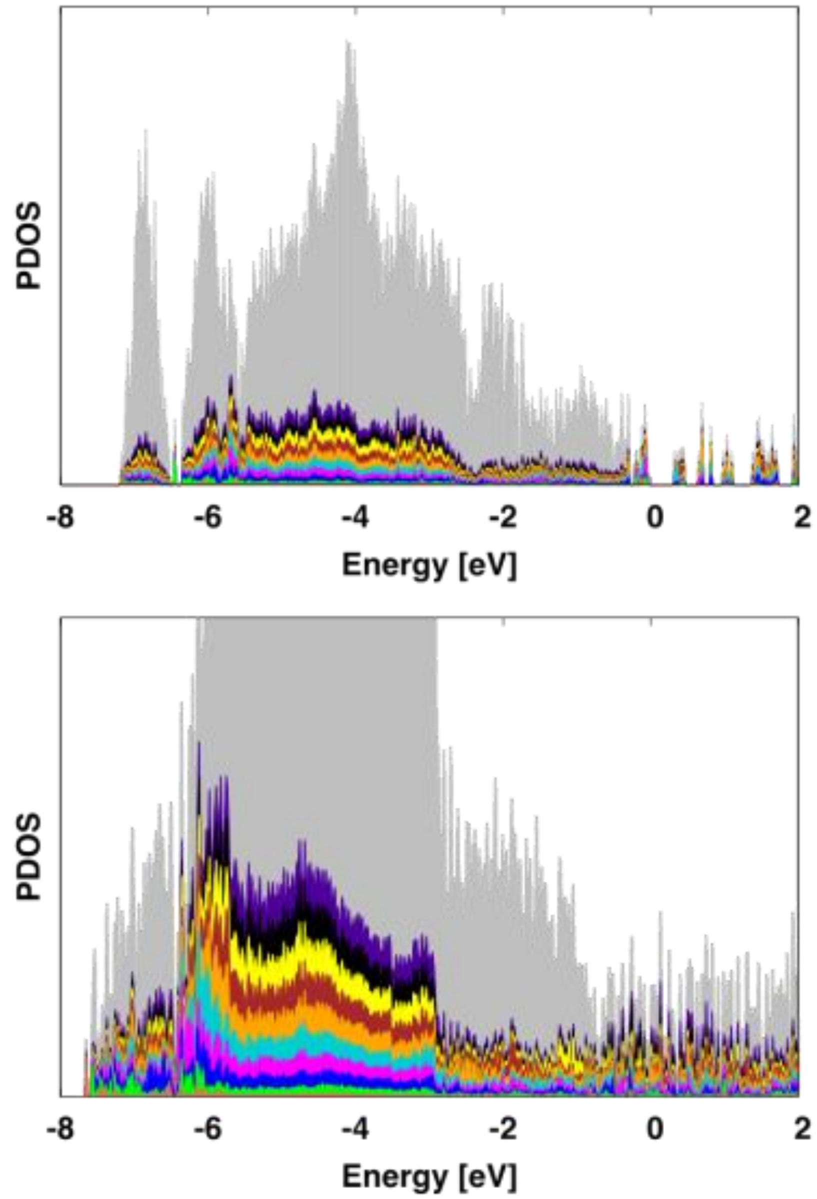

Supplementary Figure 19. The computed electronic DOS of Ag_{136} (top) and Ag_{374} (bottom) in full scale for the occupied ($E < 0$) and early unoccupied ($E > 0$) states. The steep rise of the DOS around -2.5 eV (top) and close to -3 eV (bottom) marks the edge of the Ag(4d) band. The colors in the electronic DOS panels show the state projections to global angular momenta as in Figure 5b of the main text. The Fermi energy is at zero.

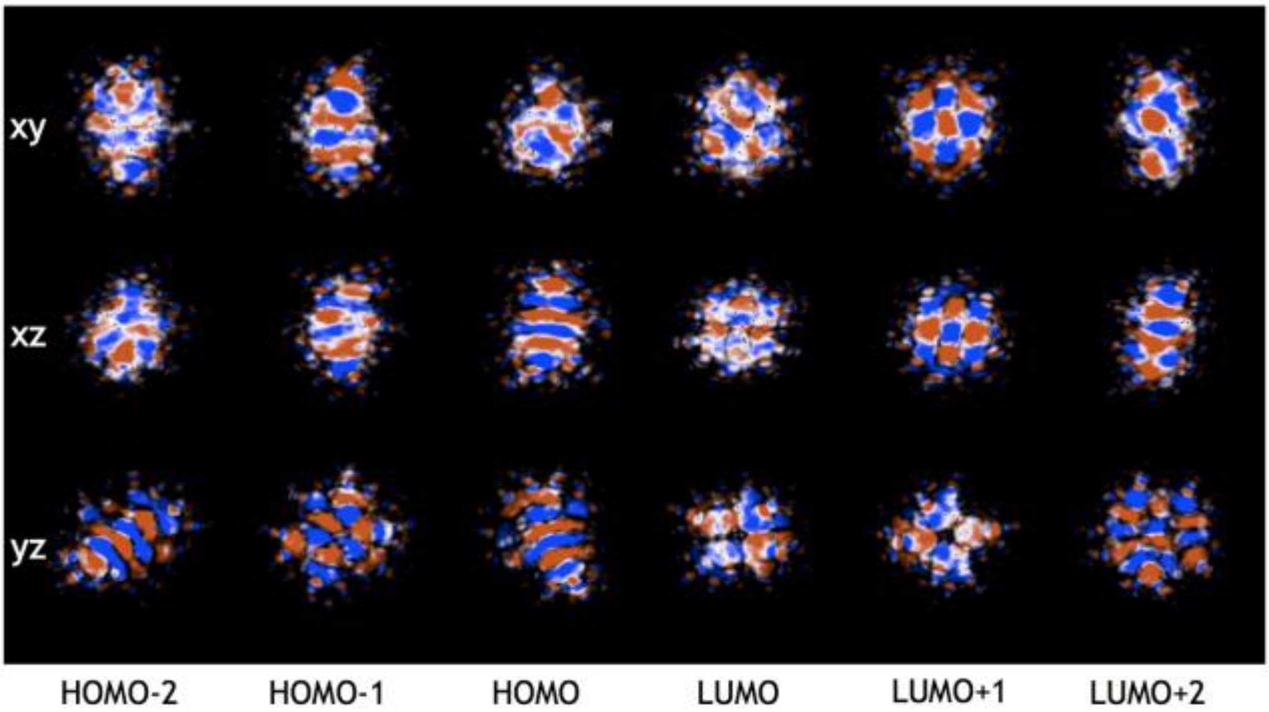

Supplementary Figure 20. The projection of the frontier orbitals (Kohn-Sham electron states) of Ag_{136} onto xy, xz and yz planes. The x-axis is along the direction of two Br atoms, and the z axis is along the longest direction of the cluster.

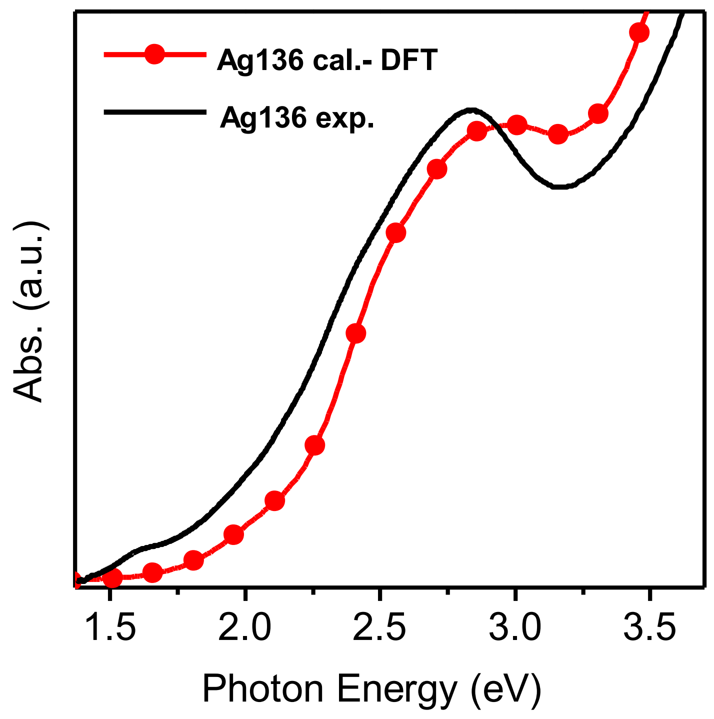

Supplementary Figure 21. Experimental and computed UV-Vis absorption spectra of Ag_{136} nanoparticles with energy units of eV.

Supplementary Table 1 Crystal data and structure refinement for $[Ag_{136}(SR)_{64}Cl_3Ag_{0.45}]\cdot PPh_4$

Identification code	Ag_{136}
Empirical formula	$C_{664}H_{852}Ag_{136}Cl_3PS_{64}Ag_{0.45}$
Formula weight	25742.63
Temperature/K	100.0(1)
Crystal system	triclinic
Space group	*P*-1
a/Å	28.8117(4)
b/Å	29.9302(4)
c/Å	54.0382(5)
α/°	79.5914(10)
β/°	86.1787(10)
γ/°	83.4260(12)
Volume/Å^3	45480.6(10)
Z	2
ρ_{calc} g/cm^3	1.885
μ/mm^{-1}	24.7
F(000)	24678.30
Crystal size/mm	0.27 × 0.07× 0.04
Radiation	Cu Kα (λ = 1.54184)
2Θ range for data collection/°	6.724 to 79.946
Index ranges	-24 ≤ h ≤ 24, -24 ≤ k ≤ 24, -45 ≤ l ≤ 45
Reflections collected	241791
Independent reflections	55043 [R_{int} = 0.0918, R_{sigma} = 0.0589]
Data/restraints/parameters	37306/1458/4478
Goodness-of-fit on *F*	1.132
Final *R* indices [I>=3σ (I)]	R_1 = 0.078, wR_2 = 0.092
Final *R* indices [all data]	R_1 = 0.099, wR_2 = 0.101
Largest diff. peak/hole / e Å^{-3}	3.95/-2.23

Supplementary Table 2 Crystal data and structure refinement for the $[Ag_{374}(SR)_{113}Br_2Cl_2]$-containing compound

Identification code	Ag_{374}
Empirical formula	$Ag_{374}C_{1130}H_{1469}Br_2Cl_2S_{113}$
Formula weight	59248.85
Temperature/K	100.01(10)
Crystal system	rhombohedral
Space group	*R*-3*c*
a/Å	67.3343(6)
b/Å	67.3343(6)
c/Å	188.5858(17)
α/°	90
β/°	90
γ/°	120
Volume/Å^3	740478(15)
Z	18
ρ_{calc} g/cm^3	2.39
μ/mm-1	36.40
F(000)	498971
Crystal size/mm	0.22 × 0.16 × 0.04
Radiation	CuKα (λ = 1.54184)
2Θ range for data collection/°	6.734 to 80
Index ranges	-58 ≤ h ≤ 61, -57 ≤ k ≤ 61, -157 ≤ l ≤ 126
Reflections collected	287815
Independent reflections	62535 [R_{int} = 0.0865, R_{sigma} = 0.0675]
Data/restraints/parameters	62536/24038/7465
Goodness-of-fit on F^2	1.261
Final R indices [I>=2σ (I)]	R_1 =0.098, wR_2 = 0.287
Final R indices [all data]	R_1 =0.148, wR_2 = 0.346
Largest diff. peak/hole / e Å^{-3}	5.49/-2.83

Supplementary Discussion.

The assignment of the apical atom sites of Ag_{136} as Cl was based on the observed electron density which was far lower than observed for the comparable apical sites in the Ag_{374} structure which have been modelled as Br the presence of which is confirmed by mass spec. In final refinement stages, a site consistent with, for example, a sulfur atom which lies adjacent to the core and for which no evidence of a phenyl group or indeed space for such a group exists. Initial models early in the refinement had included this site as the sulfur of a SPh*t*Bu ligand but this was incorrect. This mis-assignment arose from the proximity of the counter ion with its associated phenyl groups. This site was included in the final model as a chloride based on fit to the electron density, an analysis of the site geometry and the locus of the cation being immediately adjacent (phenyl groups of the cation lie interdigitated within the outer *t*BuPh shell). Synthesis of Ag_{136} using PPh_4Cl instead of the bromide salt readily yields the same material. The position of this site in the linking band between the two Ag_{30} capping structures and adjacent to the cation seems to fit the proposal that the site is quite likely a chloride, the presence of which in the product was demonstrated. A similarly puzzling site was encountered in the Ag_{374} structure which has also been modelled as chloride (one general position site per asymmetric unit - two per molecule). Alternative models have been explored, but the position of the site, again in a region adjacent to one of the Ag_{30} caps, the fit to the electron density and the resultant geometry suggest that chloride is the correct species for the site.

An additional site modelled as 45% occupancy silver is included in the Ag_{136} model. The site lies between an inner 130º+ angle Ag_2S_5 linker and the outer spanning Ag_6S_5. The final geometry in this region is mostly in accord with this proposal except for a single sulfur site of the outermost spanning Ag_2S_5 group which lies too close to this site, the large displacement parameter of this sulfur site suggests that when the silver site is occupied, the sulfur could lie further away than when it is unoccupied – this is also consistent with the large displacement parameters for the *t*BuPh which may actually be disordered. Indeed, it is possible that the occupancy of this site is symptomatic of unresolved disorder in this void adjacent region of the structure and the isotropic displacement parameter value chosen for the “additional” silver modelled at the site was chosen to be sufficiently large (comparable to U_{eq} of the neighbouring atoms) to provide an upper limit value for the occupancy. It cannot be stated with certainty whether this site corresponds to less than half of the molecules having an additional silver, or whether occupancy of this site is correlated with possible absences at other sites modelled as fully occupied within this less well defined Ag_6S_5 spanned region.

In both structures there are large voids in which Fourier difference maps are featureless and the prospect that some bromide or chloride or even additional tetraphenyl

phosphonium could be present, if highly disordered, in these regions cannot be dismissed solely on crystallographic grounds. It is proposed that these voids are filled with a highly disordered mix of solvents from the crystallization process, effectively as a non-crystalline frozen solution, there being no significant peaks in the difference density maps.

A strategy where geometric restraints (Supplementary Ref. 1) were applied to the thiolate group refinement parameters was adopted in both refinements to supplement the observed data (which is profoundly dominated by the large metal core contribution to the diffraction pattern) thereby facilitating refinement of a model including parameters for all atoms of the thiolate ligands to fit the available data in a physically and chemically sensible manner. For a small number of void adjacent groups, the minimization function gave shifts in a small number of parameters in the final refinement cycles which are larger than usual for a typical "small molecule" refinement, but these structures are not typical small molecules and such shifts for these parameters were considered to be acceptable in preference to application of stronger restraints.

Considerable challenges were encountered in refining these structures as they do not lie clearly in either the macromolecular or "small molecule" regimes. It has been necessary to use the crystallographic tools available in a considered and expert manner to arrive at structures which are as well resolved as the data permit. It is worth noting that different analysts may validly employ a range of strategies in seeking the best model available for the measured data – the nature of the decisions made in the analyses of these structures could in itself give rise to a separate paper.

Supplementary Methods.

Reagents: 4-*tert*-Butylthiophenol ($C_{10}H_{13}S$, purity 97%) was purchased from Alfa Aesar Chemical Reagent Co. Ltd. (Tianjin, China). Silver tetrafluoroborate ($AgBF_4$, purity 99%), triethylamine ($C_6H_{15}N$, purity 99%) Sodium borohydride ($NaBH_4$, purity 98%), dichloromethane (CH_2Cl_2, A.R.), chloroform ($CHCl_3$, A.R.) and methanol (CH_3OH, A.R.) were purchased from Sinopharm Chemical Reagent Co. Ltd. (Shanghai, China). The water used in all experiments was ultrapure. All reagents were used as received without further purification.

X-ray single-crystal analyses: The diffraction data of the single crystals grown from the solutions of Ag_{136} and Ag_{374} nanoparticles were collected on an Agilent Technologies SuperNova system X-ray single-crystal diffractometer with Cu Kα radiation (λ = 1.54184 Å) at 100 K. The data were processed using CrysAlis^{Pro} (Supplementary Ref. 2). The structure of Ag_{374} was solved using ShelXS (Supplementary Ref. 3) and refined using Full-matrix least-squares based on F^2 using a combination of ShelXT (Supplementary Ref. 4), ShelXS

in Olex2 (Supplementary Ref. 5) and Shelxle (Supplementary Ref. 6) and Ag_{136} was solved using ShelXL and refined using Full-matrix least-squares based on *F* in CRYSTALS (Supplementary Ref. 7). Detailed crystal data and structure refinement results for both compounds are given in Supplementary Tables 1 and 2. Careful application of the electron density contouring facility in Shelxle was employed to examine the phenyl and *t*Bu groups and “donuts” of electron density (of varying resolution but self-evidently phenyl density) were seen in all modelled phenyl cases. The extent to which the individual atomic sites could be discerned varied in a rationalizable manner around the molecules and groups having less distinct atom sites correspond to those which have larger refined displacement ellipsoids. Similar variation in the approximately conical densities of the *t*Bu groups was observed for all thiolates included in the model.

Some evidence (an observed systematic bias where $F_o > F_c$ in particular zones for the least well fitted data) of twinning is observed in the Ag_{136} refinement and the largest twin component identified in a ROTAX (Supplementary Ref. 8) analysis was refined clearly accounting for the largest spurious electron density peaks appearing within the core if twinning is not included in the model.

Supplementary References.

1. Watkin, D. J. *Acta. Cryst.*, **A50**, 411-437(1994).

2. CrysAlis[Pro] Version 1.171.35.19. (2011). Agilent Technologies Inc. Santa Clara, CA, USA.

3. Sheldrick, G. M. SHELXT - Integrated space-group and crystal-structure determination. *Acta Cryst.* **C71**, 3-8 (2015).

4. Sheldrick, G. M. A short history of SHELX. *Acta Cryst*. **A64**, 112-122 (2008)..

5. Dolomanov et al. OLEX2: a complete structure solution, refinement and analysis program. *J. Appl. Cryst*. **42**, 339-341 (2009).

6. Hübschle, C. B., Sheldrick G. M. & Dittrich B. ShelXle: a Qt graphical user interface for SHELXL *J. Appl. Cryst.*, **44**, 1281-1284 (2011).

7. Cooper, R. I., Gould, R. O., Parsons, S., Watkin. D. J. The derivation of non-merohedral twin laws during refinement by analysis of poorly fitting intensity data and the refinement of non-merohedrally twinned crystal structures in the program. *J. Appl. Crystallogr.* **35**,168-174 (2002).

8. Betteridge, P.W., Carruthers, J.R., Cooper, R.I., Prout, K. & Watkin, D.J. CRYSTALS version 12: software for guided crystal structure analysis. *J. Appl. Cryst.* **36**,1487(2003).
